# Supplementary figures and images for: PARP14 is regulated by the PARP9/DTX3L complex and promotes interferon γ-induced ADP-ribosylation
Source: EMBO J. 2024 Jun 4;43(14):6. doi: 10.1038/s44318-024-00125-1 (PMC11251048; doi:10.1038/s44318-024-00125-1)

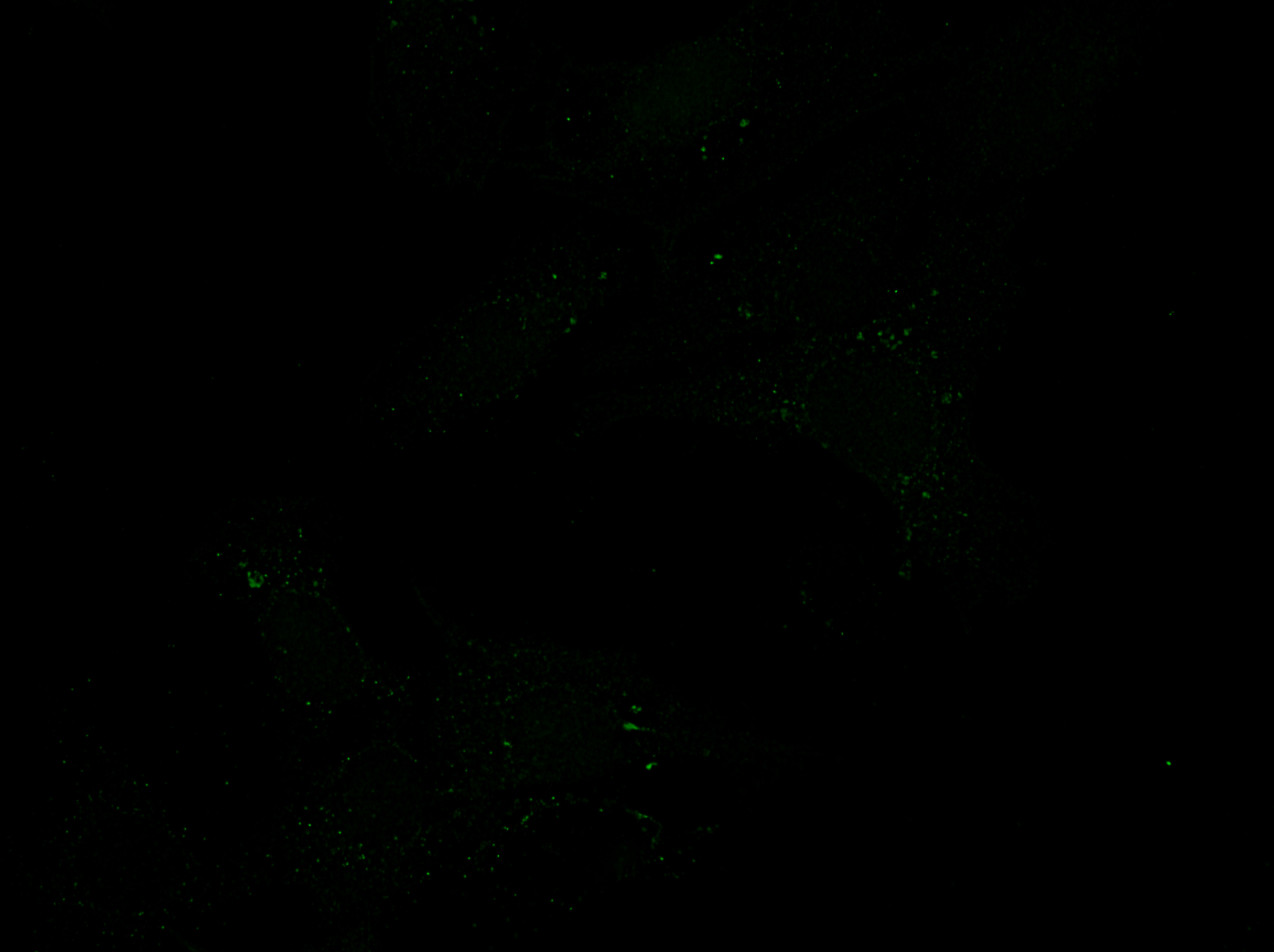

Supplement: Supplementary file 2 — Source data Fig. 1 [file 44318_2024_125_MOESM2_ESM.zip › Figure 1 - Source Data/1A/SOURCE DATA 1A A549 C eAf1521-568 monoADPrHCA354-488 A488.tif]

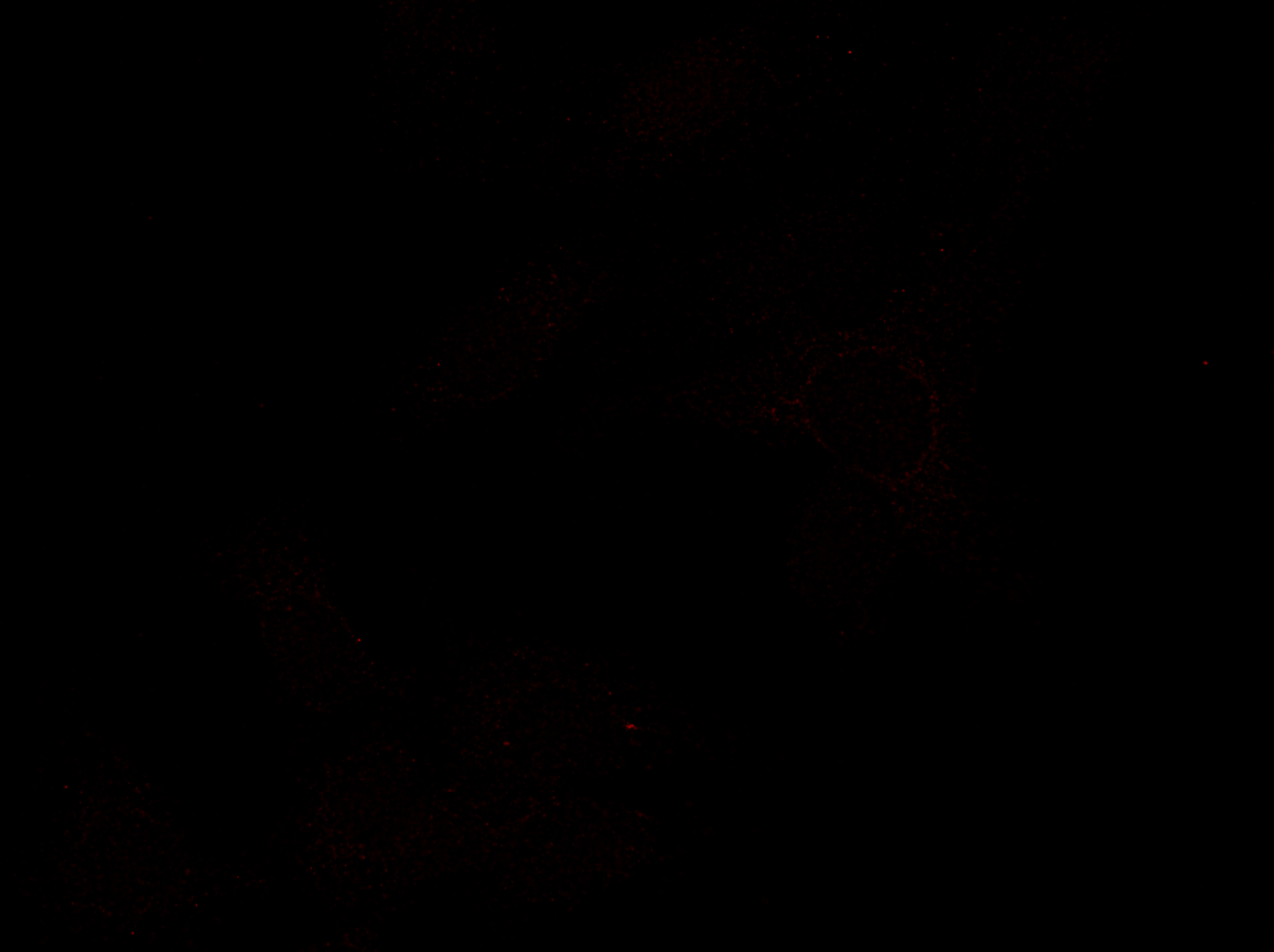

Supplement: Supplementary file 2 — Source data Fig. 1 [file 44318_2024_125_MOESM2_ESM.zip › Figure 1 - Source Data/1A/SOURCE DATA 1A A549 C eAf1521-568 monoADPrHCA354-488 A568.tif]

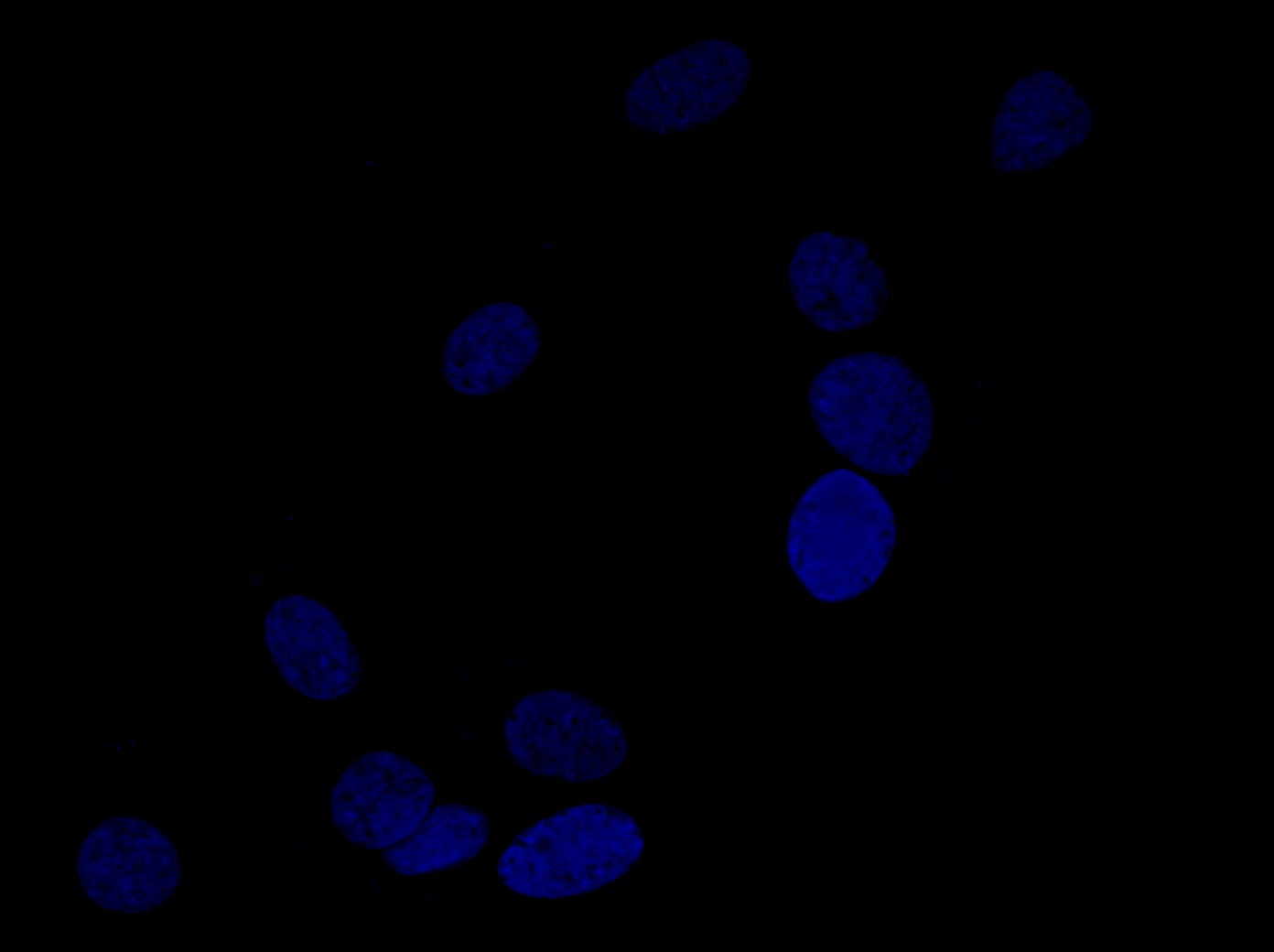

Supplement: Supplementary file 2 — Source data Fig. 1 [file 44318_2024_125_MOESM2_ESM.zip › Figure 1 - Source Data/1A/SOURCE DATA 1A A549 C eAf1521-568 monoADPrHCA354-488 DAPI.tif]

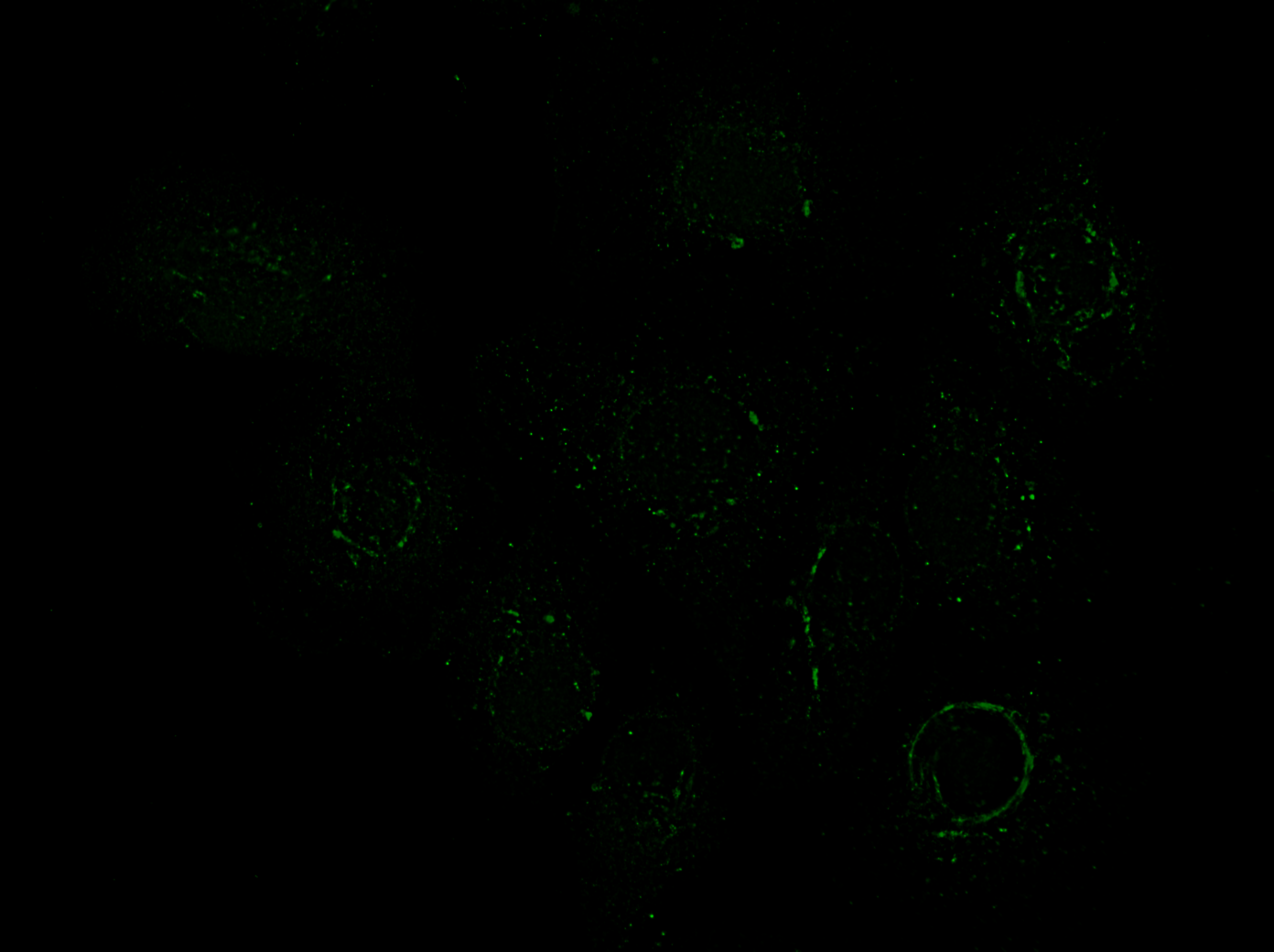

Supplement: Supplementary file 2 — Source data Fig. 1 [file 44318_2024_125_MOESM2_ESM.zip › Figure 1 - Source Data/1A/SOURCE DATA 1A A549 IFN eAf1521-568 monoADPrHCA354-488 A488.tif]

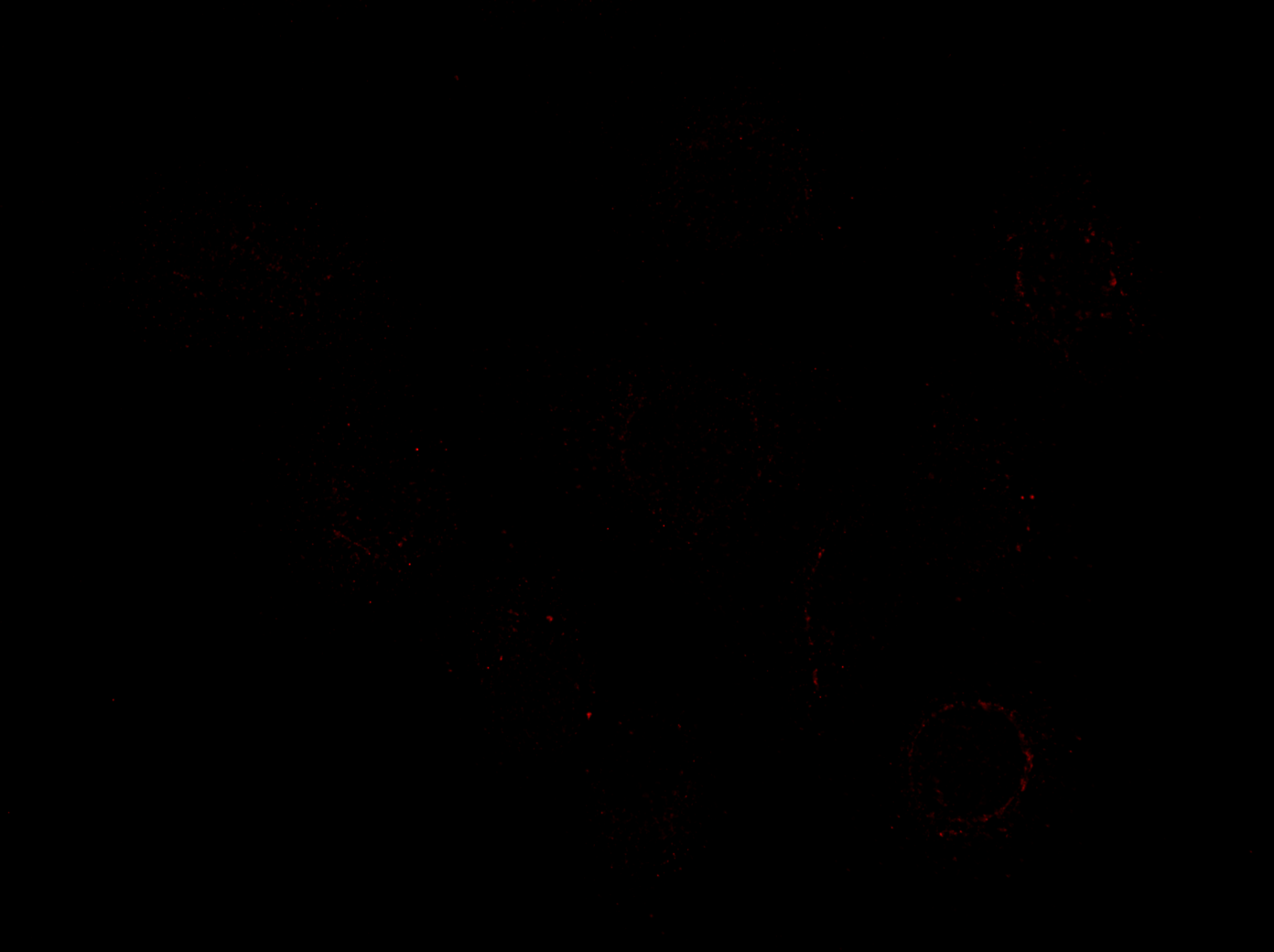

Supplement: Supplementary file 2 — Source data Fig. 1 [file 44318_2024_125_MOESM2_ESM.zip › Figure 1 - Source Data/1A/SOURCE DATA 1A A549 IFN eAf1521-568 monoADPrHCA354-488 A568.tif]

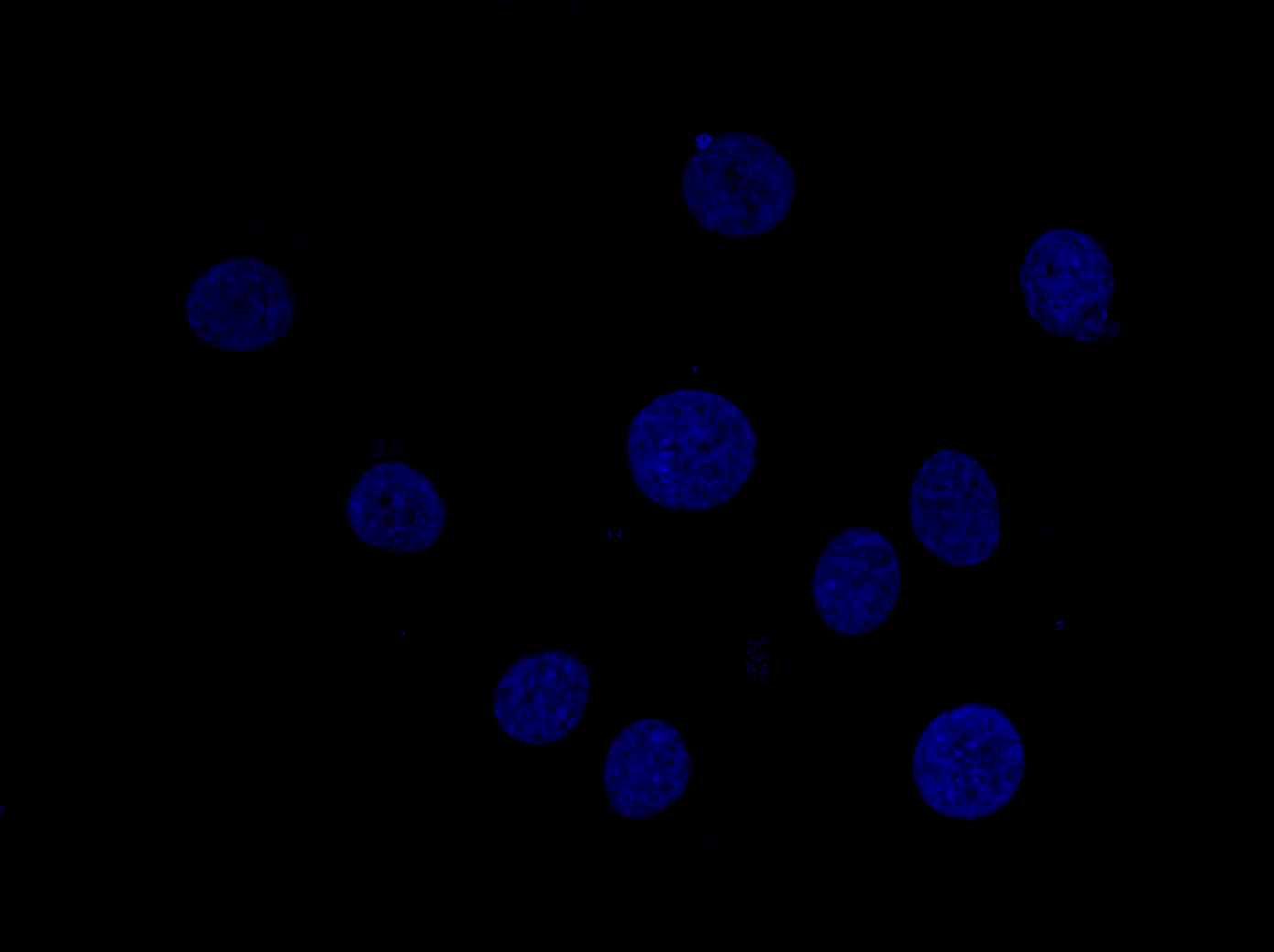

Supplement: Supplementary file 2 — Source data Fig. 1 [file 44318_2024_125_MOESM2_ESM.zip › Figure 1 - Source Data/1A/SOURCE DATA 1A A549 IFN eAf1521-568 monoADPrHCA354-488 DAPI.tif]

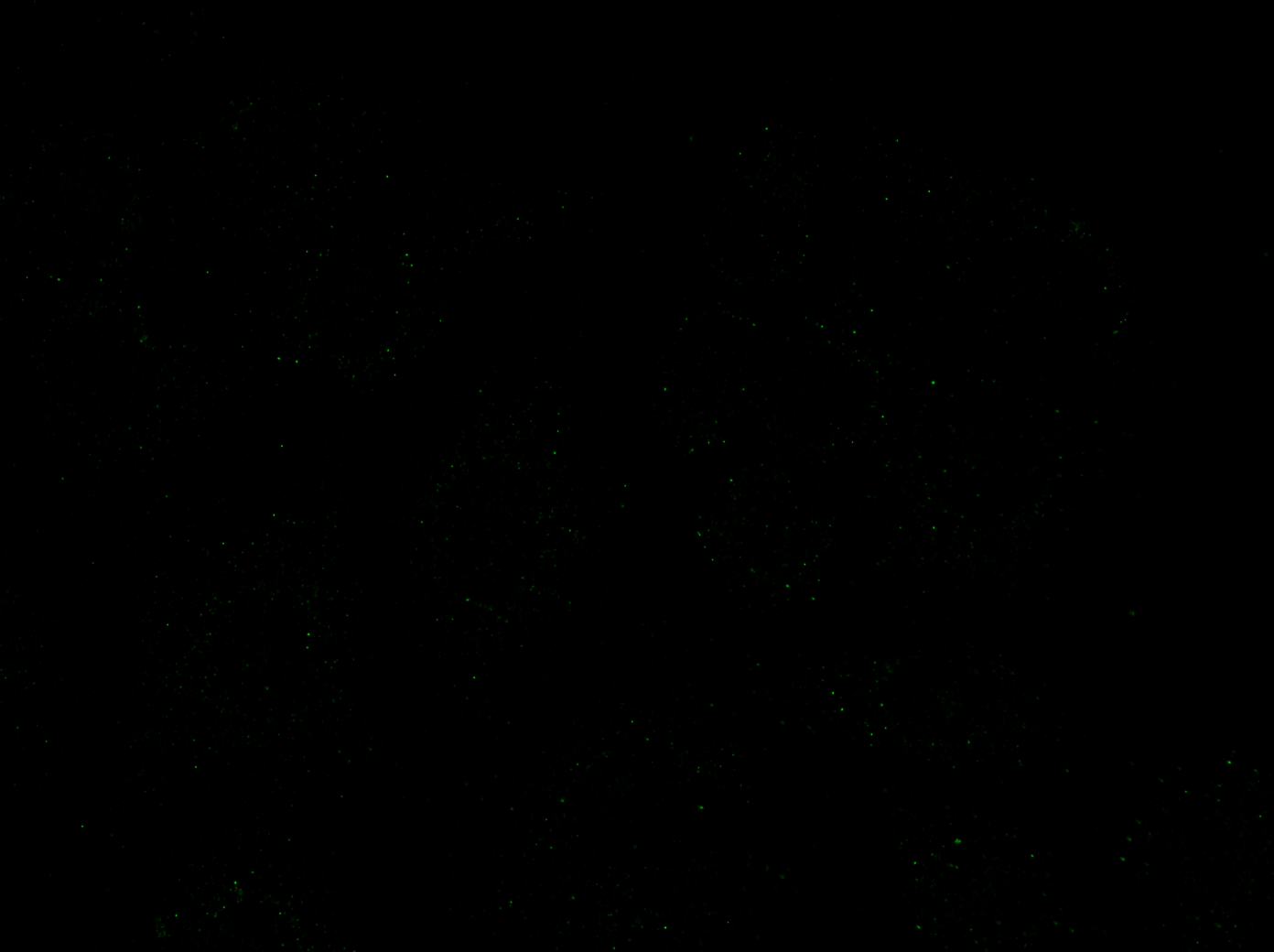

Supplement: Supplementary file 2 — Source data Fig. 1 [file 44318_2024_125_MOESM2_ESM.zip › Figure 1 - Source Data/1B/SOURCE DATA 1B A549 C eAf1521-568 monoADPrHCA355-488 A488.tif]

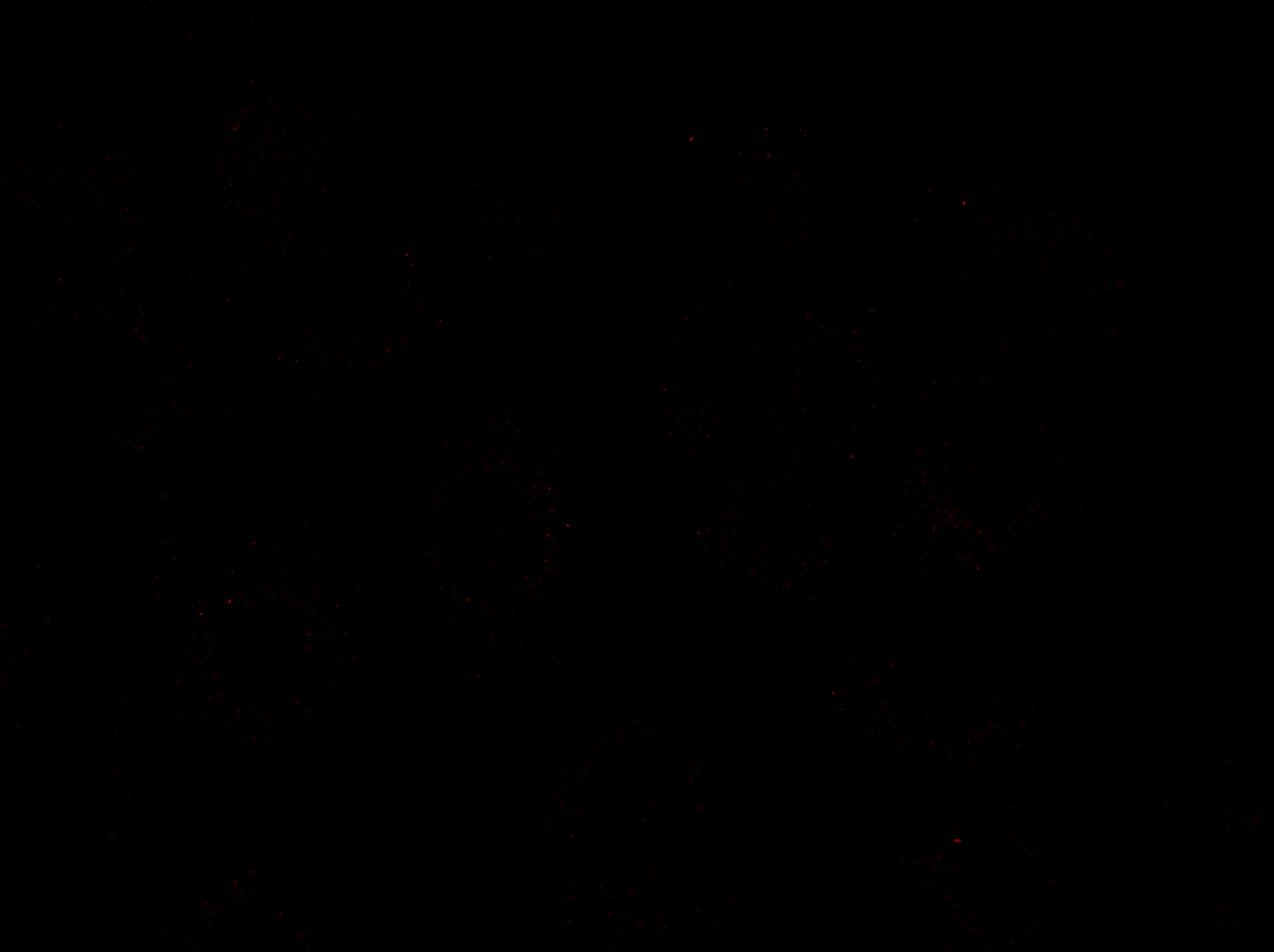

Supplement: Supplementary file 2 — Source data Fig. 1 [file 44318_2024_125_MOESM2_ESM.zip › Figure 1 - Source Data/1B/SOURCE DATA 1B A549 C eAf1521-568 monoADPrHCA355-488 A568.tif]

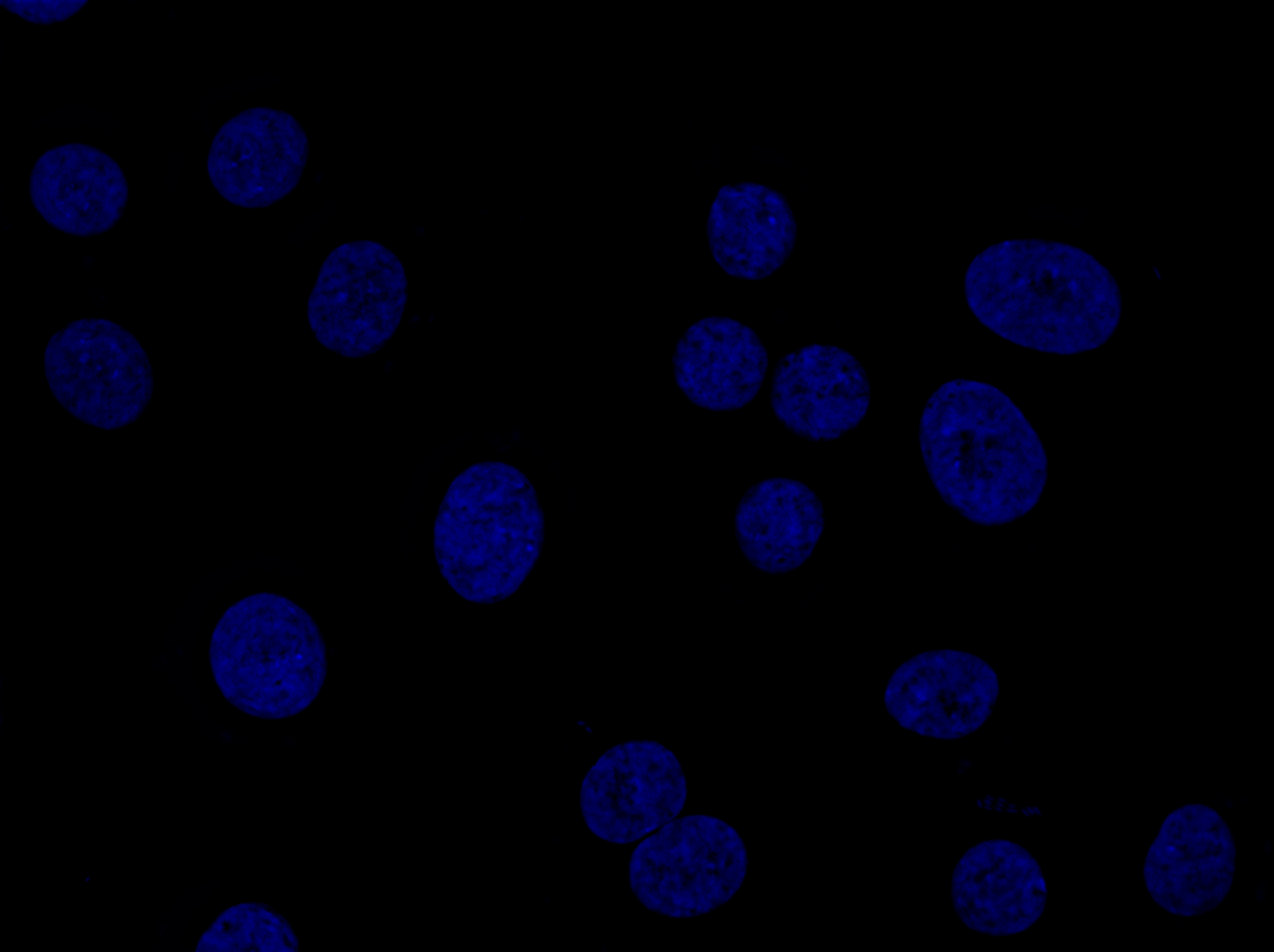

Supplement: Supplementary file 2 — Source data Fig. 1 [file 44318_2024_125_MOESM2_ESM.zip › Figure 1 - Source Data/1B/SOURCE DATA 1B A549 C eAf1521-568 monoADPrHCA355-488 DAPI.tif]

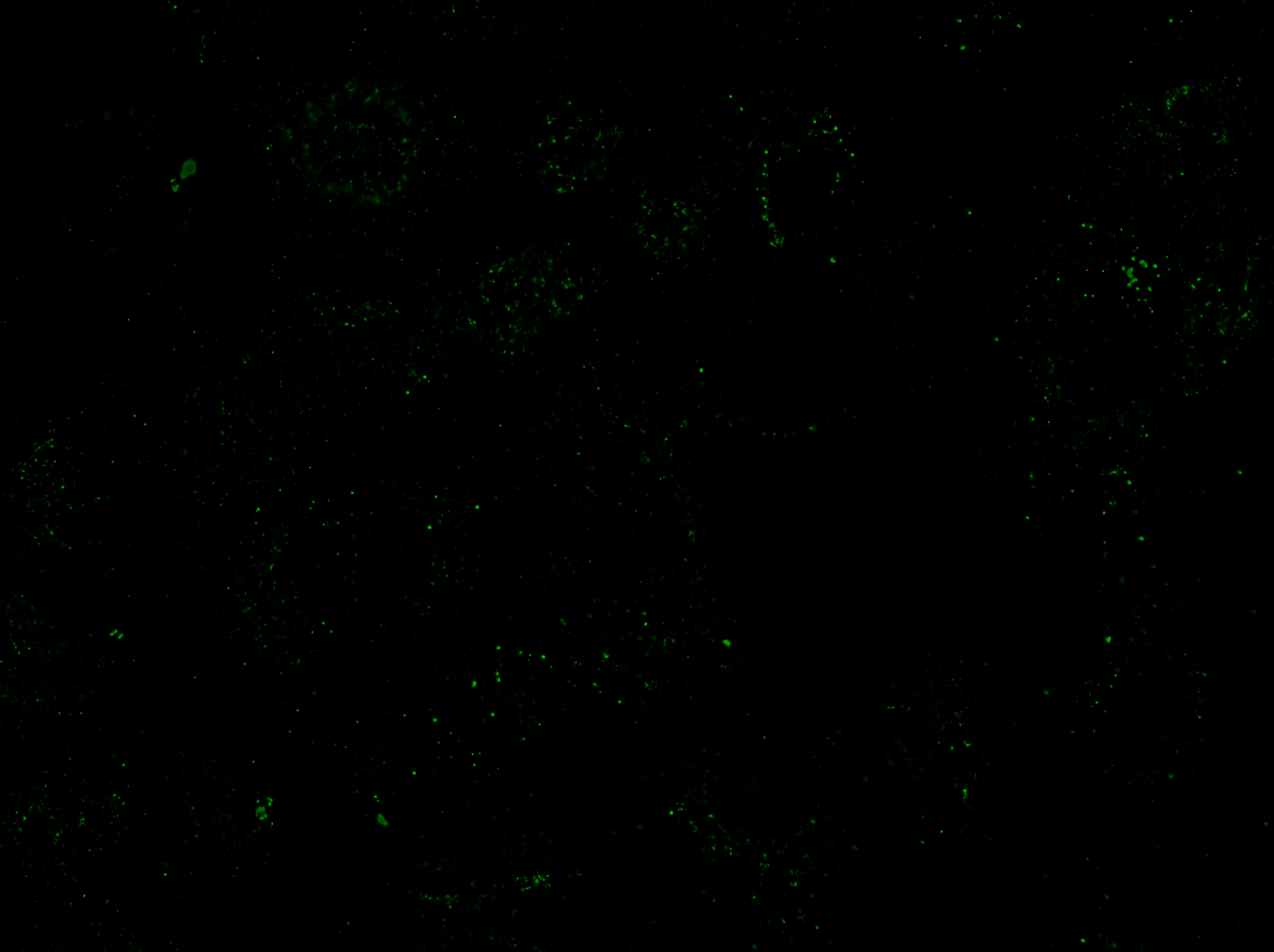

Supplement: Supplementary file 2 — Source data Fig. 1 [file 44318_2024_125_MOESM2_ESM.zip › Figure 1 - Source Data/1B/SOURCE DATA 1B A549 IFN eAf1521-568 monoADPrHCA355-488 A488.tif]

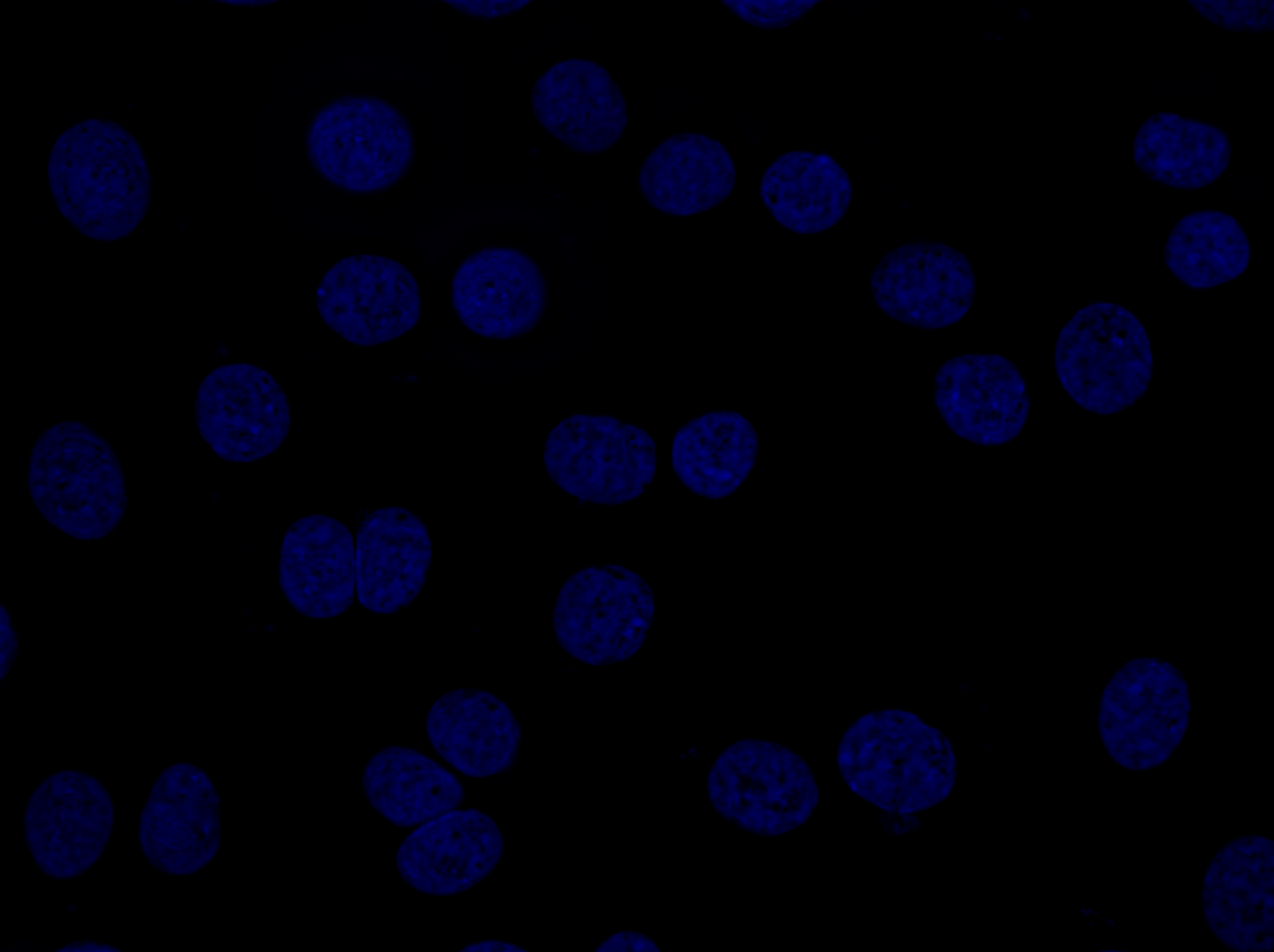

Supplement: Supplementary file 2 — Source data Fig. 1 [file 44318_2024_125_MOESM2_ESM.zip › Figure 1 - Source Data/1B/SOURCE DATA 1B A549 IFN eAf1521-568 monoADPrHCA355-488 A568 DAPI.tif]

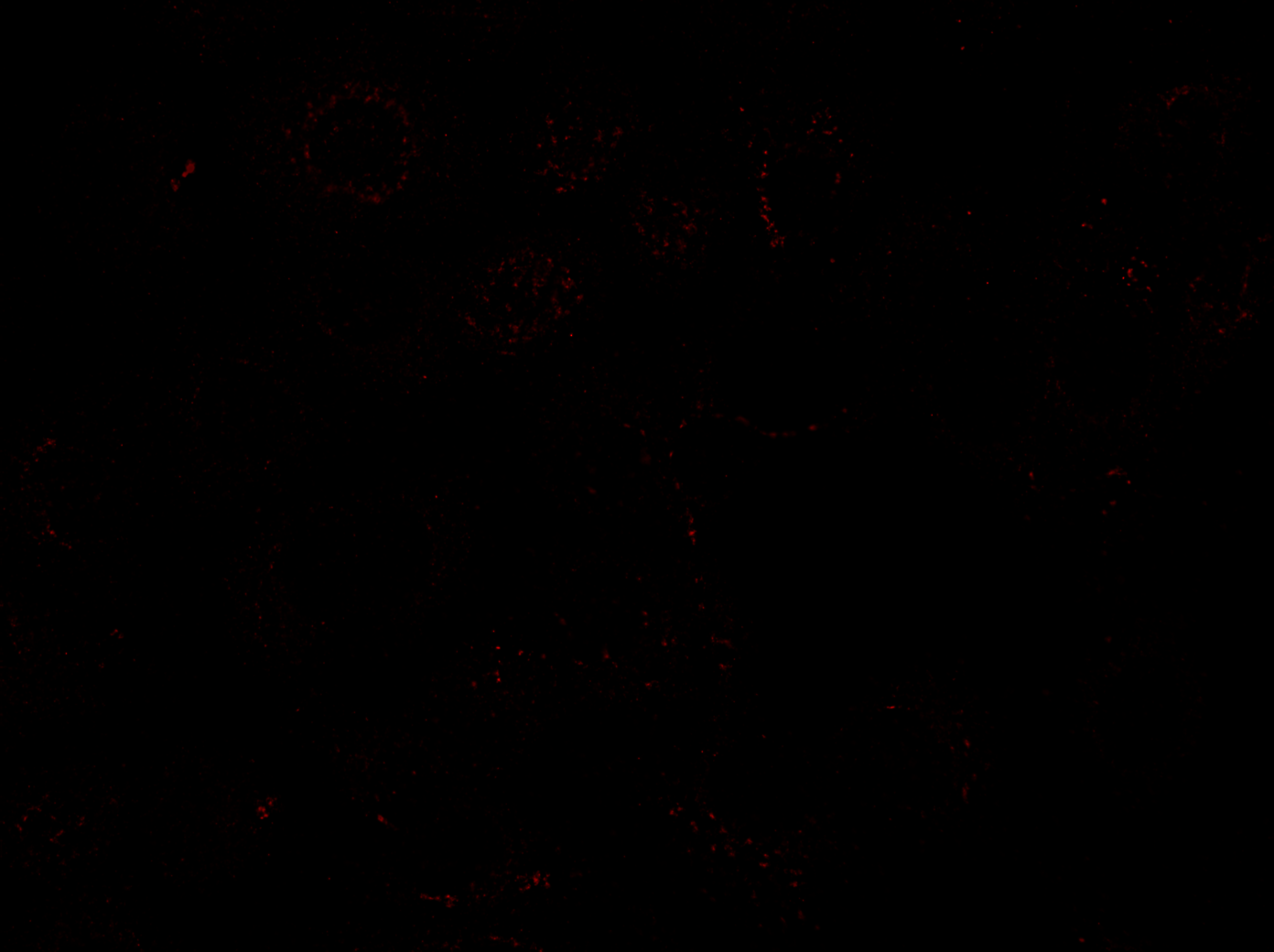

Supplement: Supplementary file 2 — Source data Fig. 1 [file 44318_2024_125_MOESM2_ESM.zip › Figure 1 - Source Data/1B/SOURCE DATA 1B A549 IFN eAf1521-568 monoADPrHCA355-488 A568.tif]

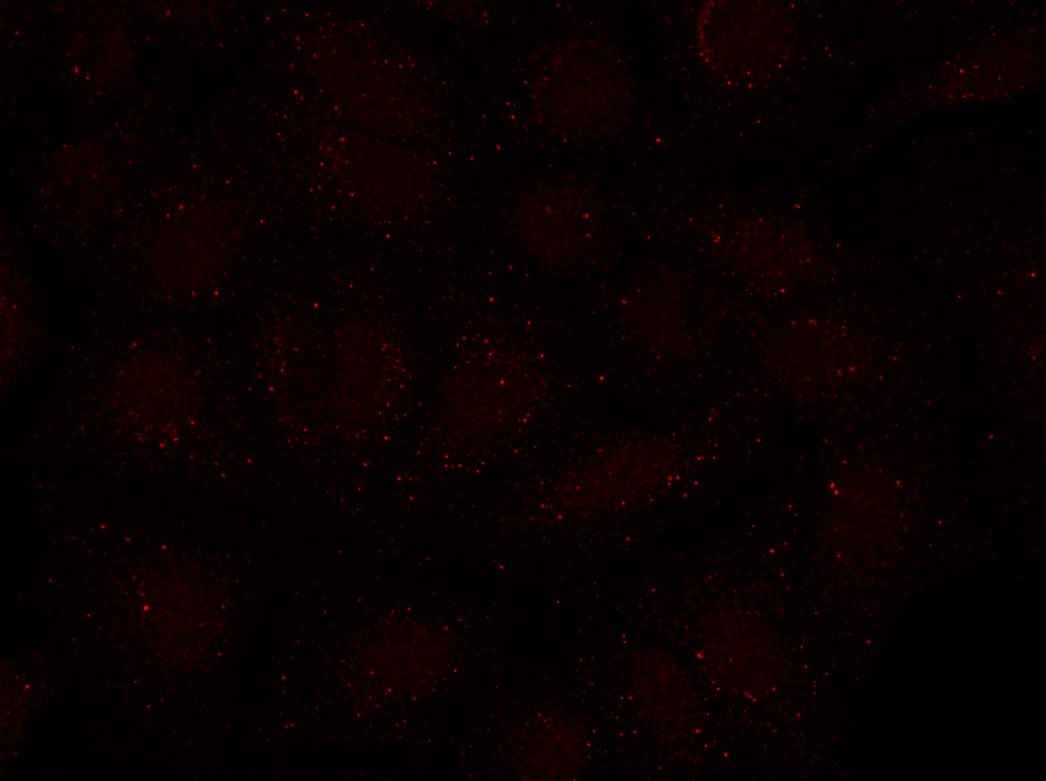

Supplement: Supplementary file 4 — Source data Fig. 3 [file 44318_2024_125_MOESM4_ESM.zip › Figure 3 - Source Data/3A/C - PARP14 x ADPr - ADPr.tif]

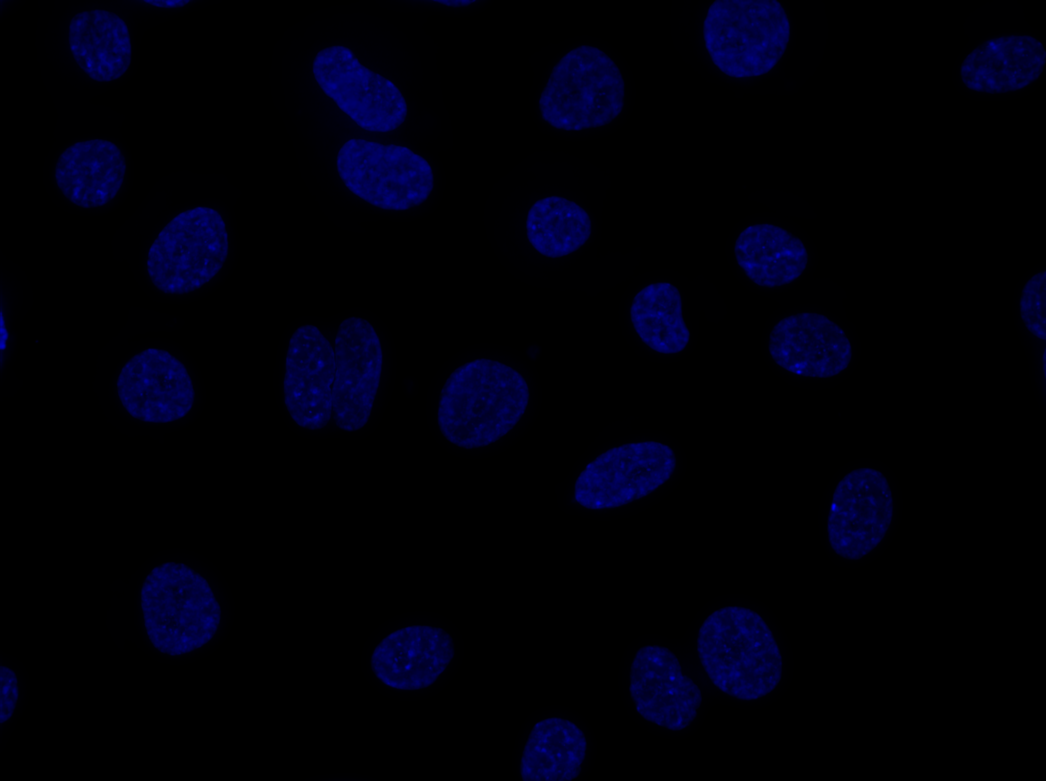

Supplement: Supplementary file 4 — Source data Fig. 3 [file 44318_2024_125_MOESM4_ESM.zip › Figure 3 - Source Data/3A/C - PARP14 x ADPr - DAPI.tif]

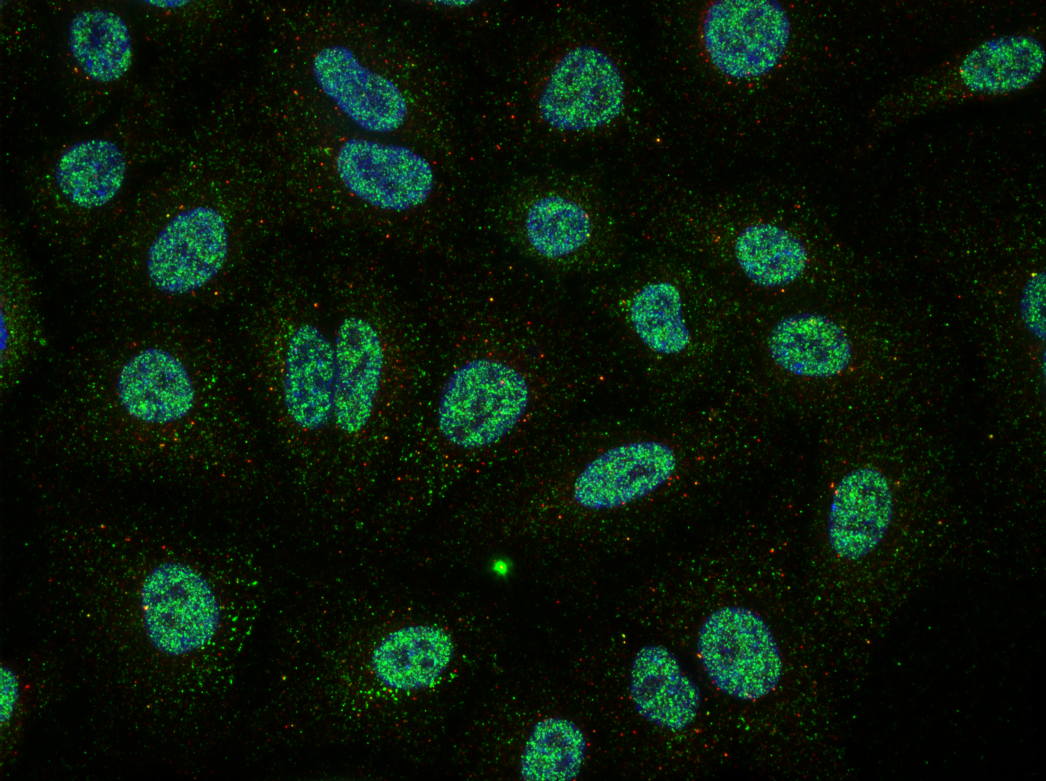

Supplement: Supplementary file 4 — Source data Fig. 3 [file 44318_2024_125_MOESM4_ESM.zip › Figure 3 - Source Data/3A/C - PARP14 x ADPr - merge.tif]

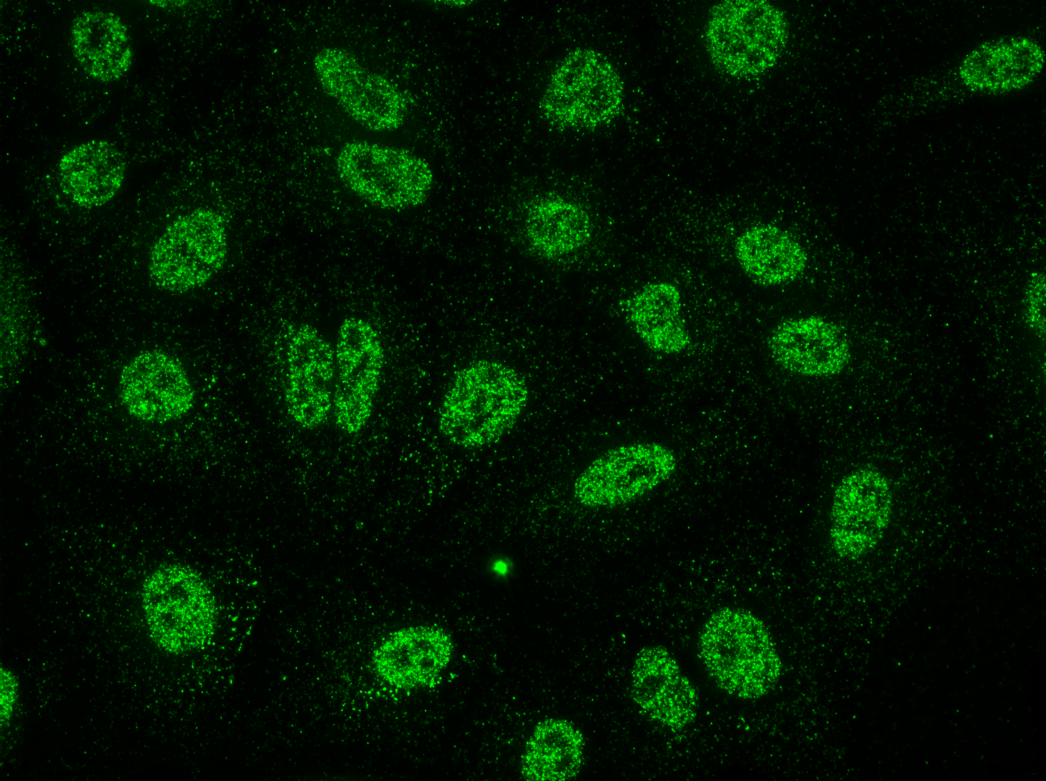

Supplement: Supplementary file 4 — Source data Fig. 3 [file 44318_2024_125_MOESM4_ESM.zip › Figure 3 - Source Data/3A/C - PARP14 x ADPr - PARP14.tif]

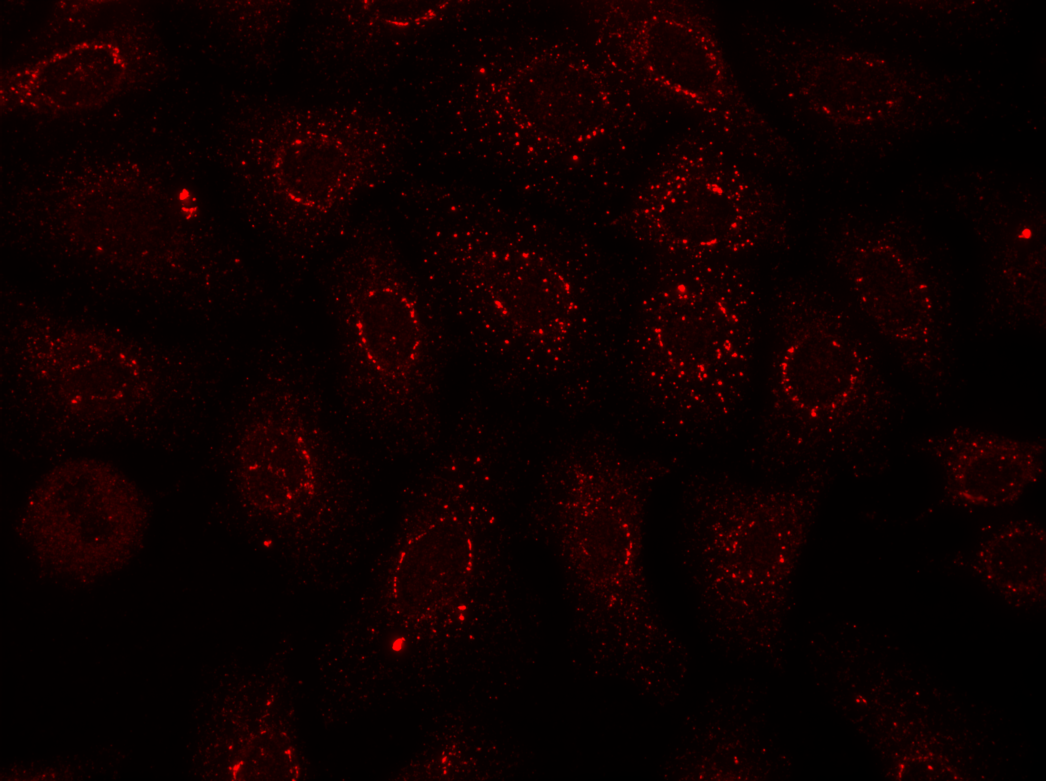

Supplement: Supplementary file 4 — Source data Fig. 3 [file 44318_2024_125_MOESM4_ESM.zip › Figure 3 - Source Data/3A/IFN - PARP14 x ADPr - ADPr.tif]

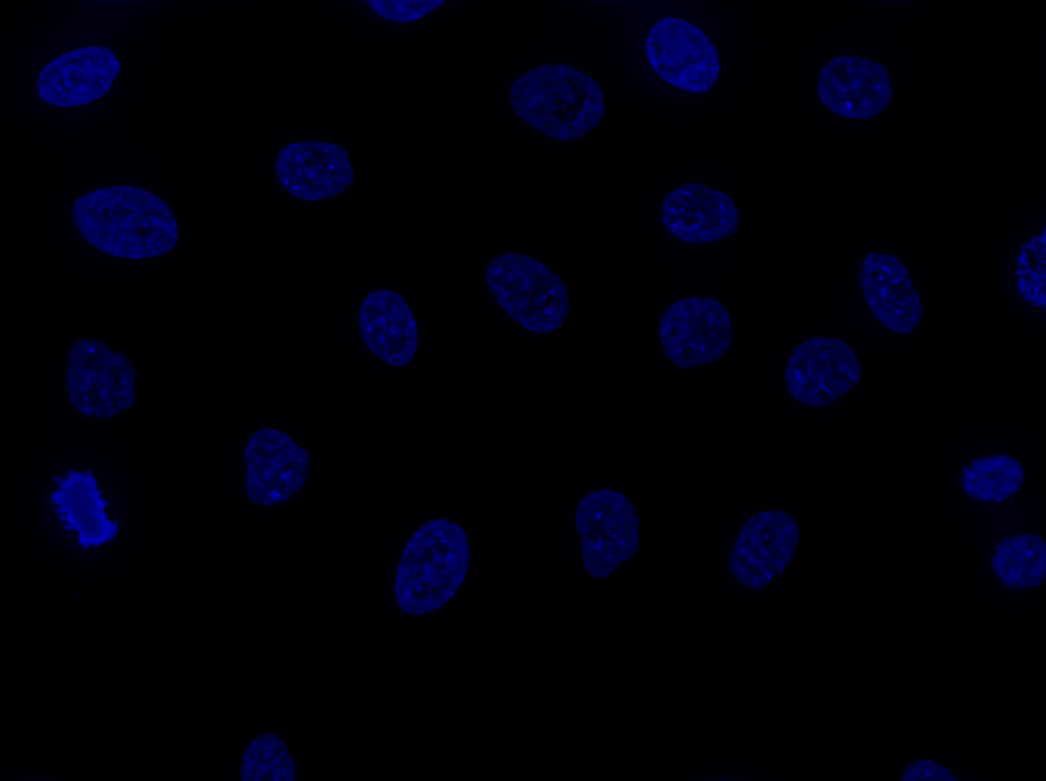

Supplement: Supplementary file 4 — Source data Fig. 3 [file 44318_2024_125_MOESM4_ESM.zip › Figure 3 - Source Data/3A/IFN - PARP14 x ADPr - DAPI.tif]

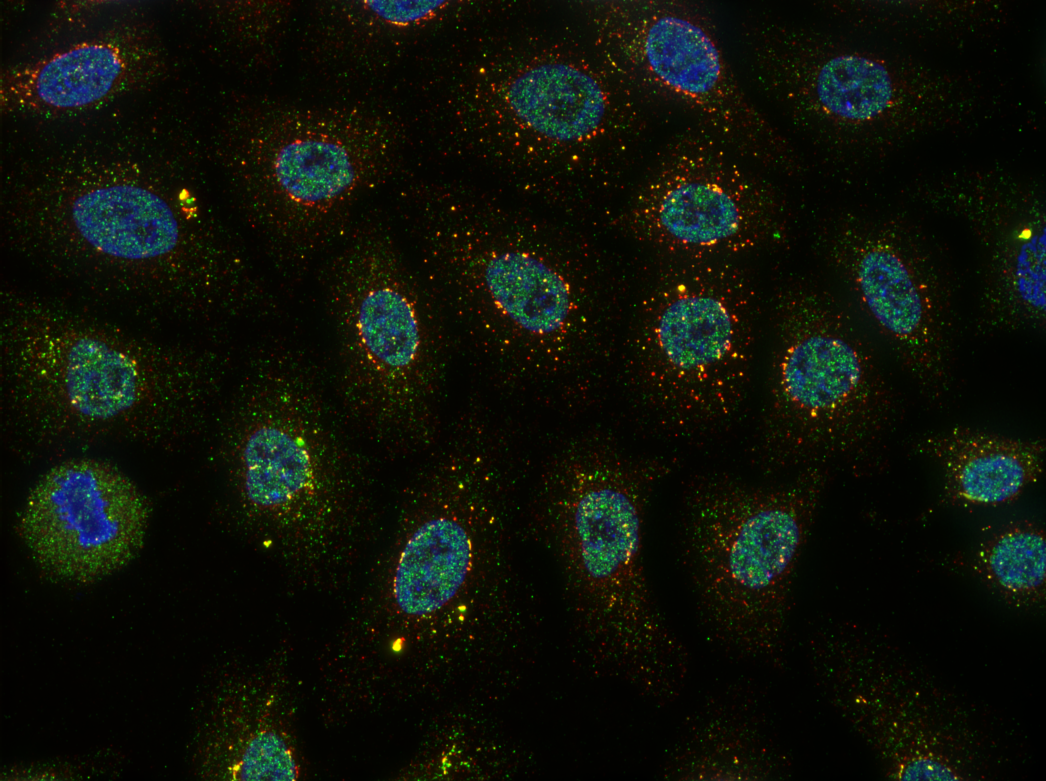

Supplement: Supplementary file 4 — Source data Fig. 3 [file 44318_2024_125_MOESM4_ESM.zip › Figure 3 - Source Data/3A/IFN - PARP14 x ADPr - merge.tif]

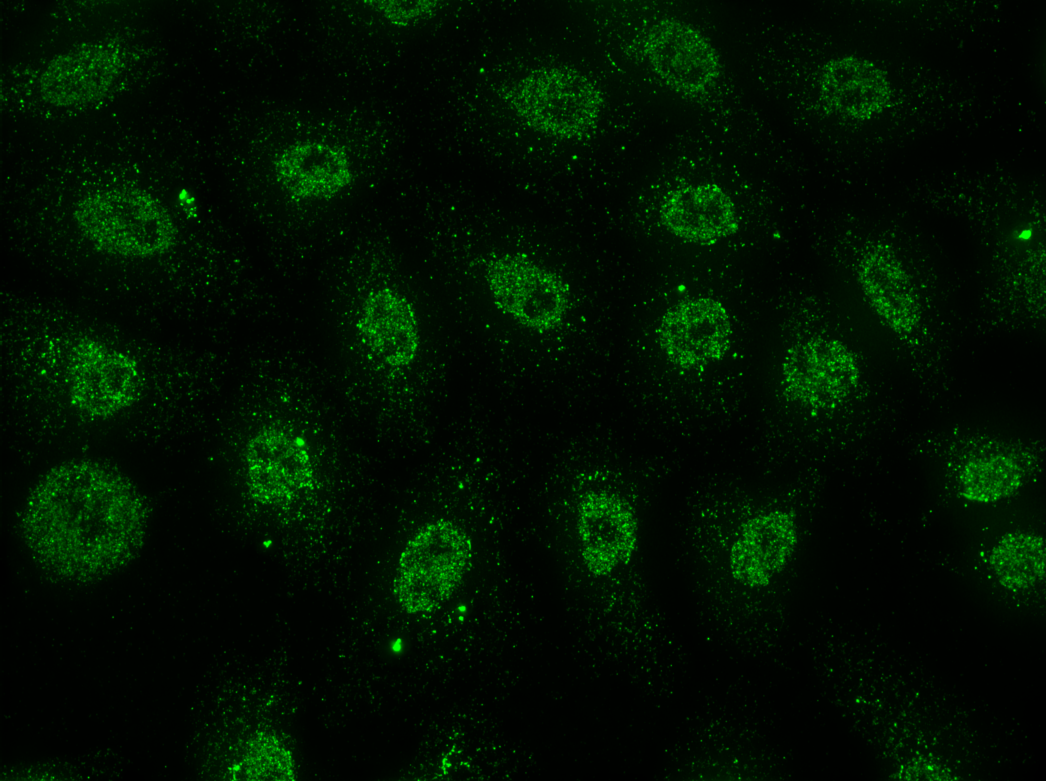

Supplement: Supplementary file 4 — Source data Fig. 3 [file 44318_2024_125_MOESM4_ESM.zip › Figure 3 - Source Data/3A/IFN - PARP14 x ADPr - PARP14.tif]

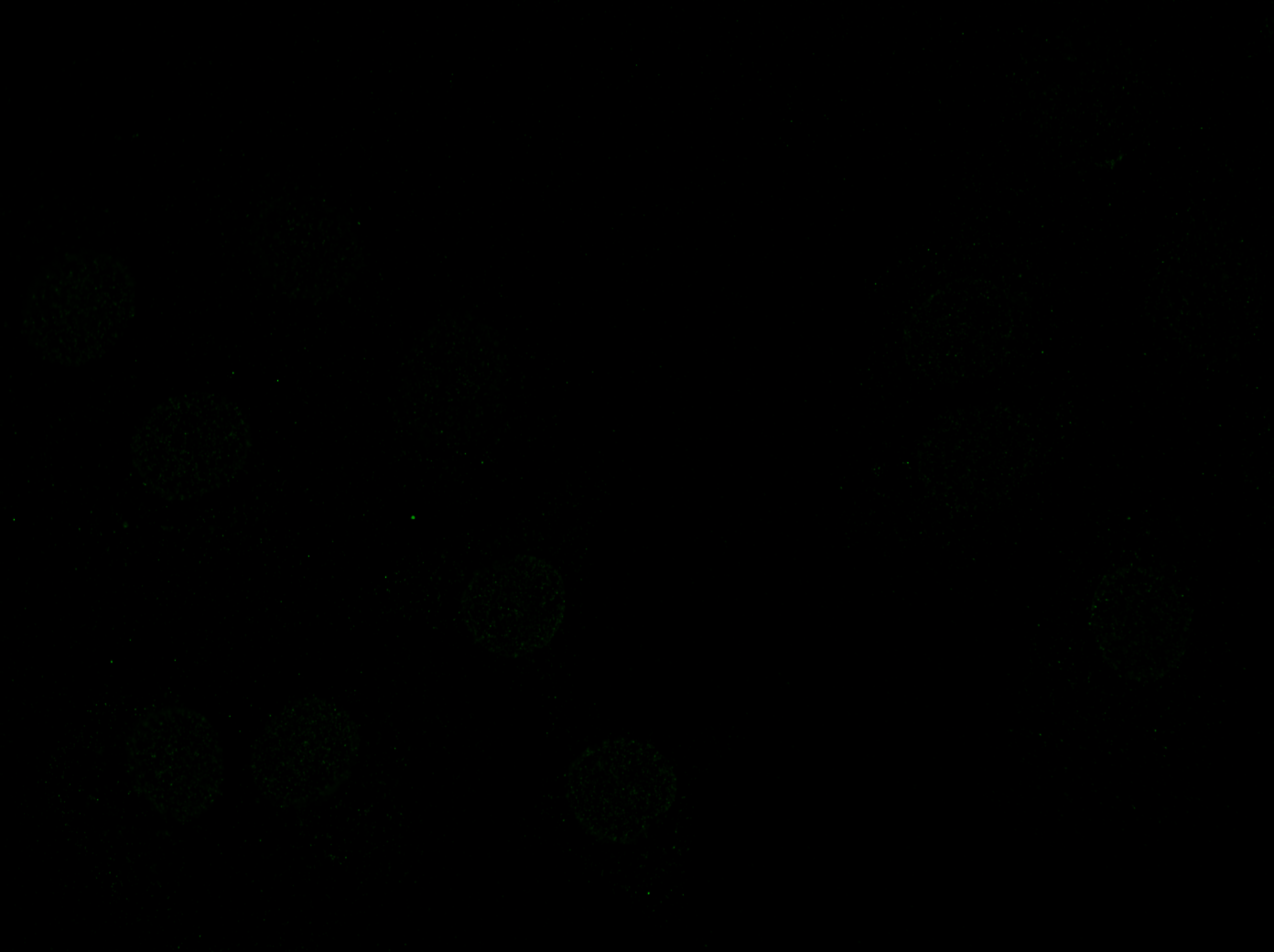

Supplement: Supplementary file 4 — Source data Fig. 3 [file 44318_2024_125_MOESM4_ESM.zip › Figure 3 - Source Data/3B/SOURCE DATA 3B A549 C ADPr-A568 PARP9-A488 A488.tif]

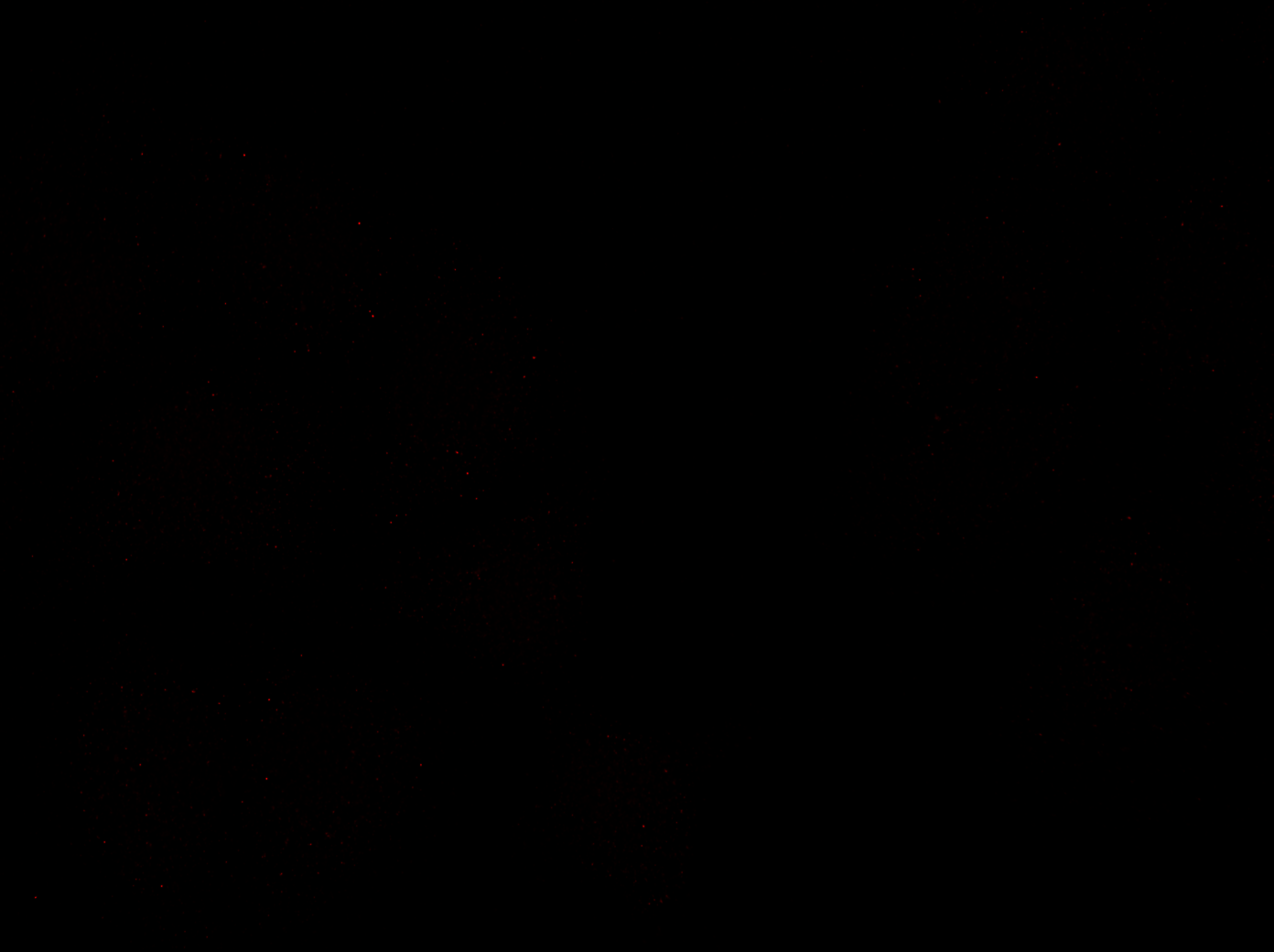

Supplement: Supplementary file 4 — Source data Fig. 3 [file 44318_2024_125_MOESM4_ESM.zip › Figure 3 - Source Data/3B/SOURCE DATA 3B A549 C ADPr-A568 PARP9-A488 A568.tif]

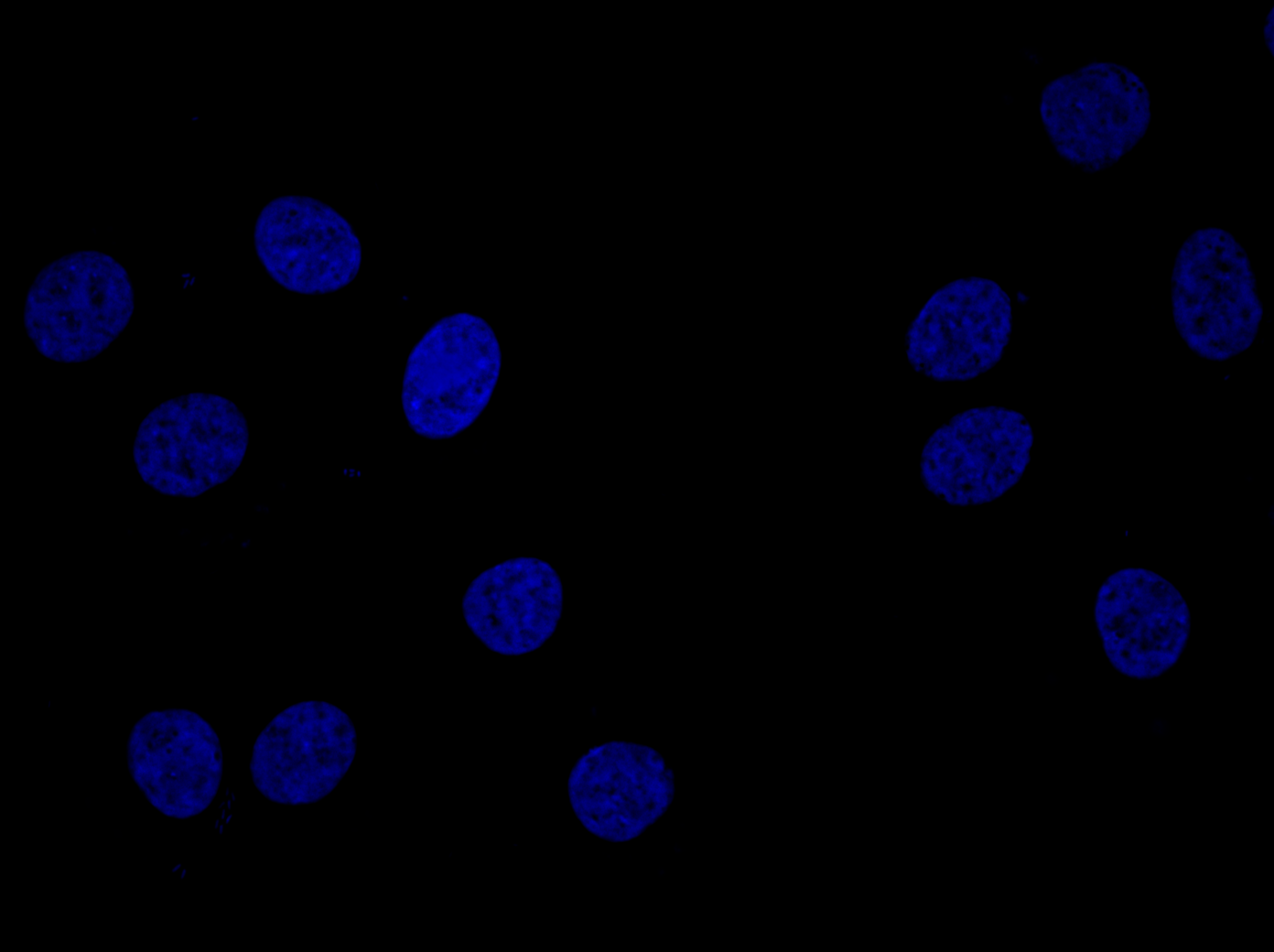

Supplement: Supplementary file 4 — Source data Fig. 3 [file 44318_2024_125_MOESM4_ESM.zip › Figure 3 - Source Data/3B/SOURCE DATA 3B A549 C ADPr-A568 PARP9-A488 DAPI.tif]

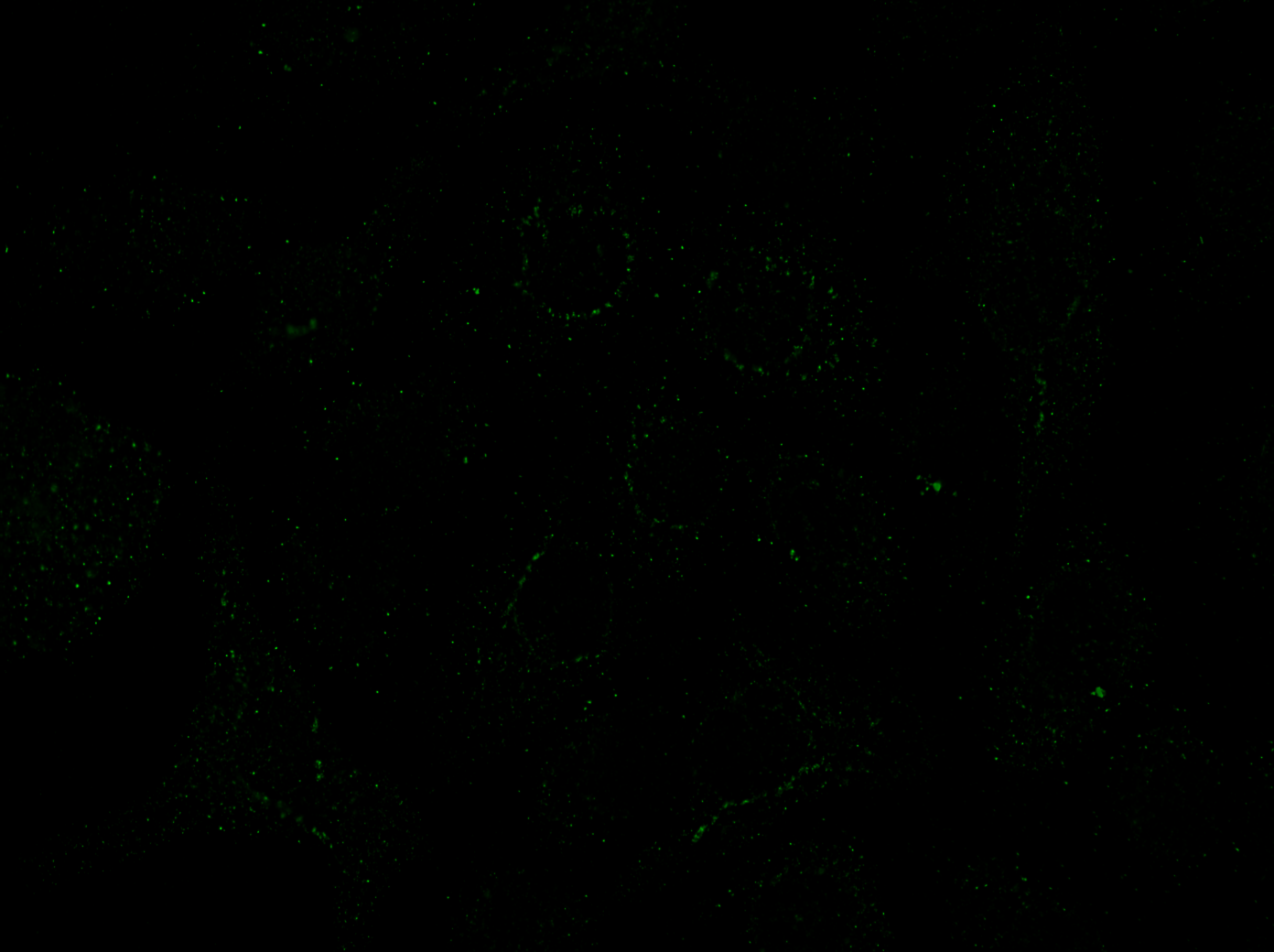

Supplement: Supplementary file 4 — Source data Fig. 3 [file 44318_2024_125_MOESM4_ESM.zip › Figure 3 - Source Data/3B/SOURCE DATA 3B A549 IFN ADPr-A568 PARP9-A488 A488.tif]

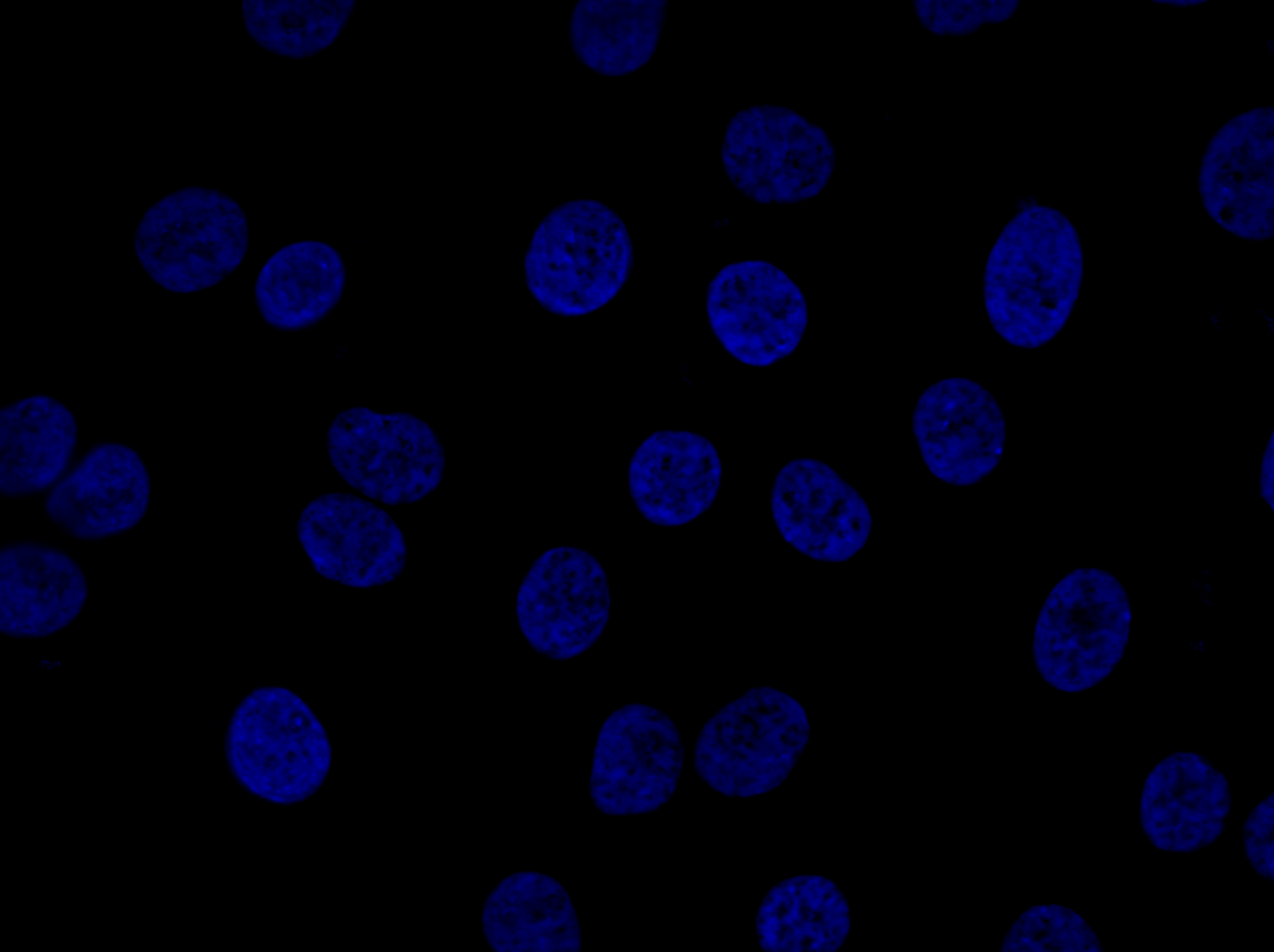

Supplement: Supplementary file 4 — Source data Fig. 3 [file 44318_2024_125_MOESM4_ESM.zip › Figure 3 - Source Data/3B/SOURCE DATA 3B A549 IFN ADPr-A568 PARP9-A488 A568 DAPI.tif]

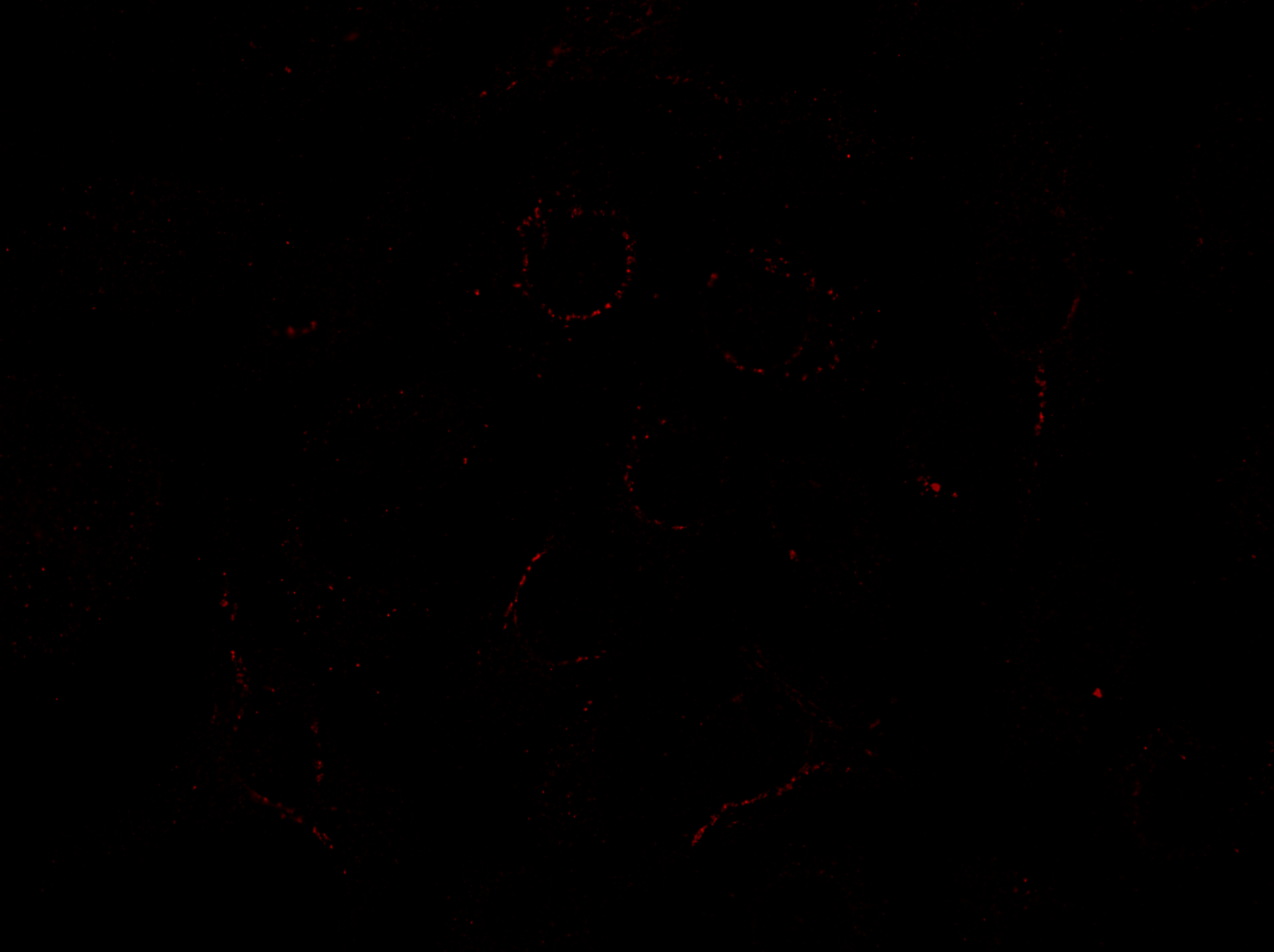

Supplement: Supplementary file 4 — Source data Fig. 3 [file 44318_2024_125_MOESM4_ESM.zip › Figure 3 - Source Data/3B/SOURCE DATA 3B A549 IFN ADPr-A568 PARP9-A488 A568.tif]

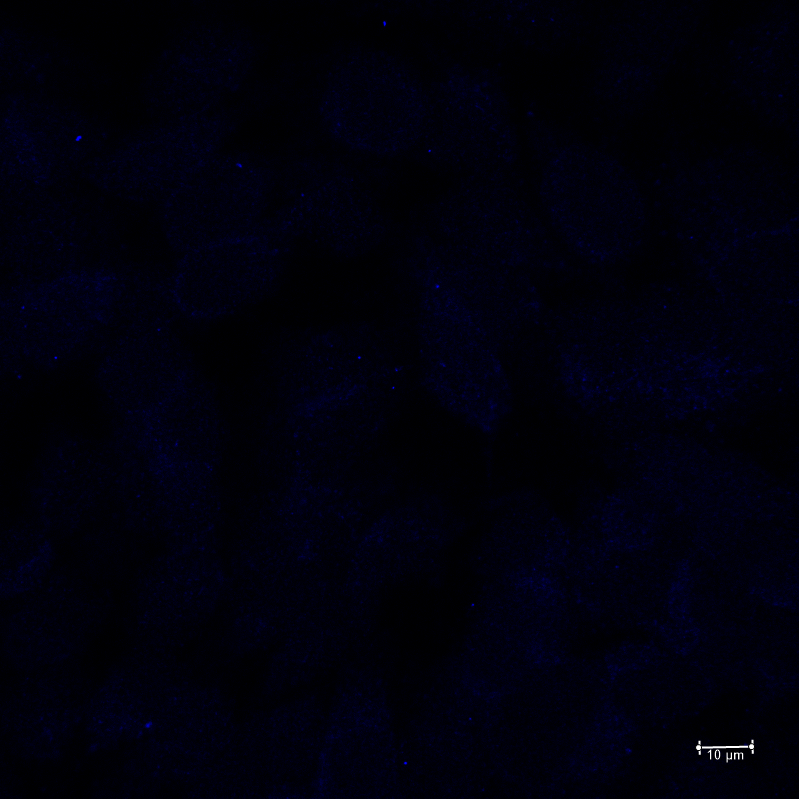

Supplement: Supplementary file 4 — Source data Fig. 3 [file 44318_2024_125_MOESM4_ESM.zip › Figure 3 - Source Data/3C/no transfected - ADPr.tif]

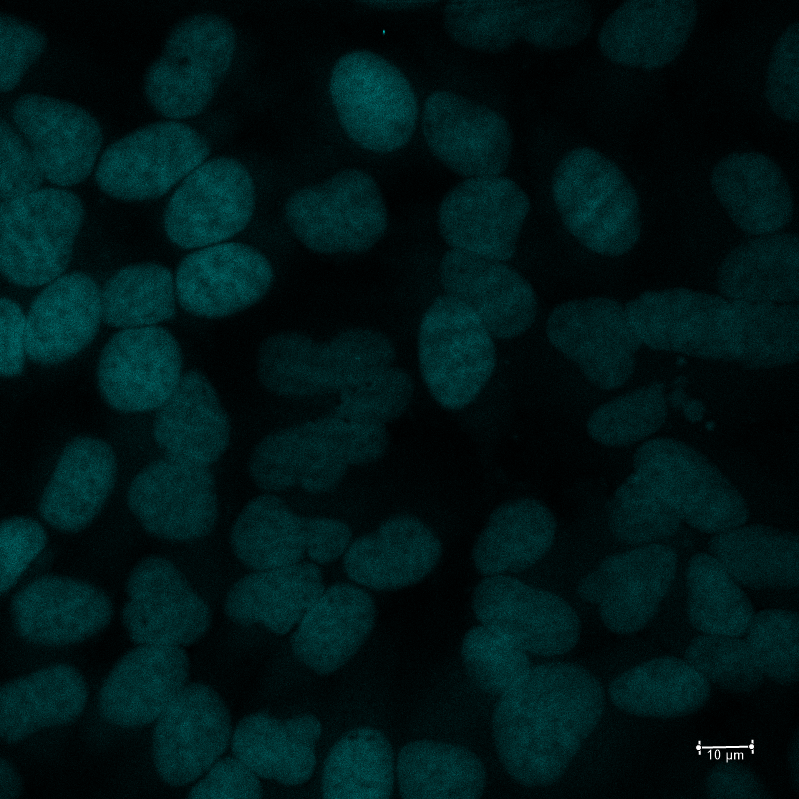

Supplement: Supplementary file 4 — Source data Fig. 3 [file 44318_2024_125_MOESM4_ESM.zip › Figure 3 - Source Data/3C/no transfected - DAPI.tif]

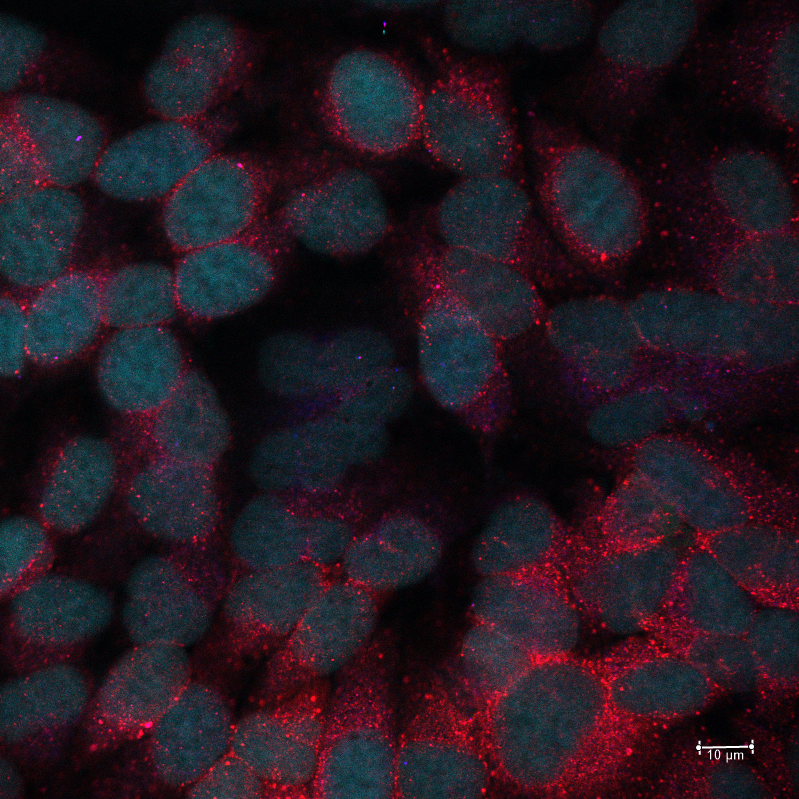

Supplement: Supplementary file 4 — Source data Fig. 3 [file 44318_2024_125_MOESM4_ESM.zip › Figure 3 - Source Data/3C/no transfected - merge.tif]

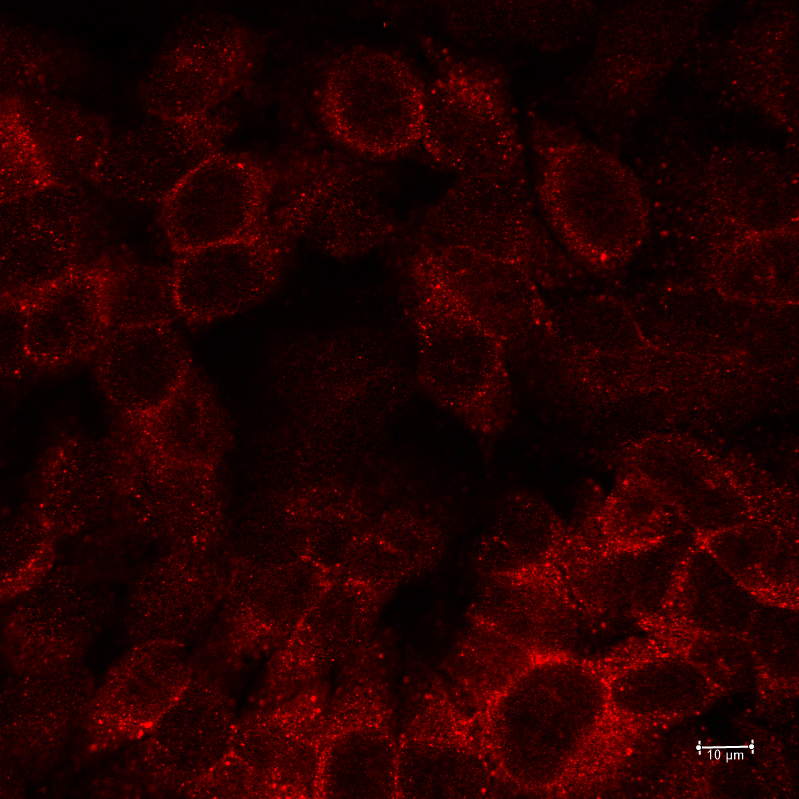

Supplement: Supplementary file 4 — Source data Fig. 3 [file 44318_2024_125_MOESM4_ESM.zip › Figure 3 - Source Data/3C/no transfected - PARP9.tif]

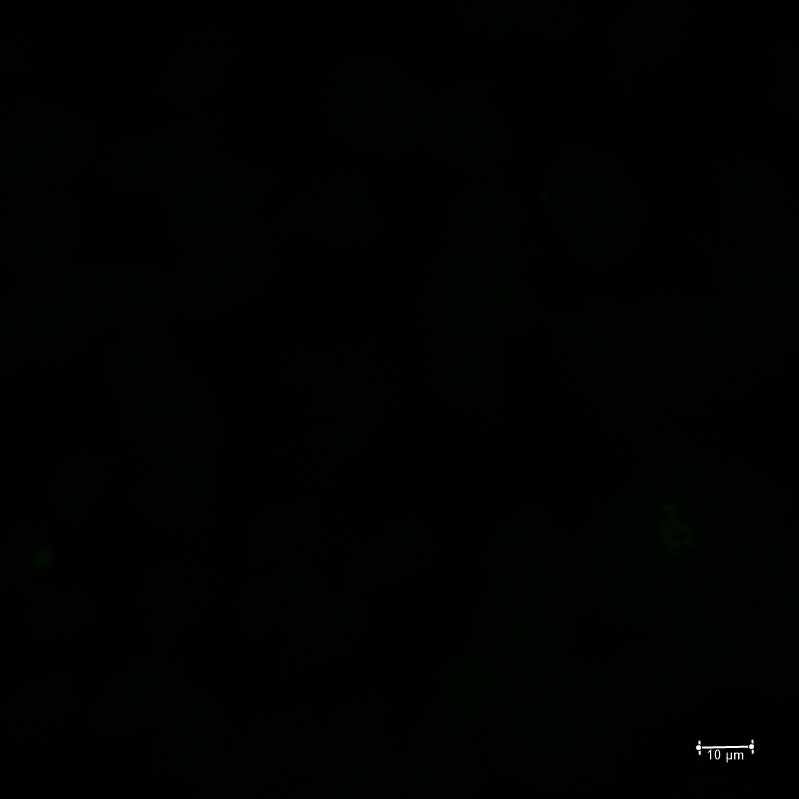

Supplement: Supplementary file 4 — Source data Fig. 3 [file 44318_2024_125_MOESM4_ESM.zip › Figure 3 - Source Data/3C/no transfected - YFP.tif]

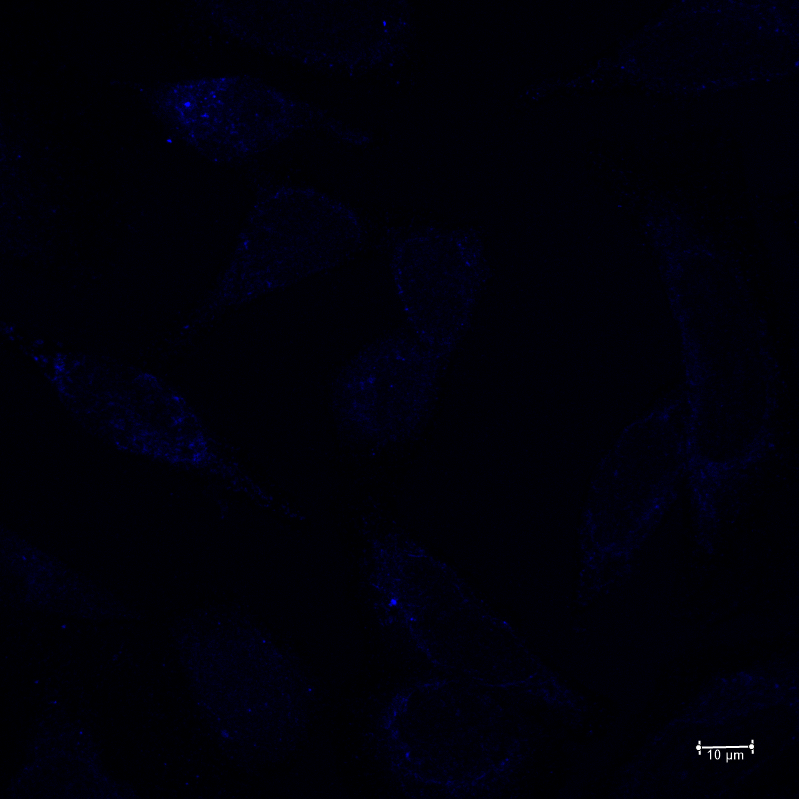

Supplement: Supplementary file 4 — Source data Fig. 3 [file 44318_2024_125_MOESM4_ESM.zip › Figure 3 - Source Data/3C/YFP-ev x PARP9 x ADPr - ADPr.tif]

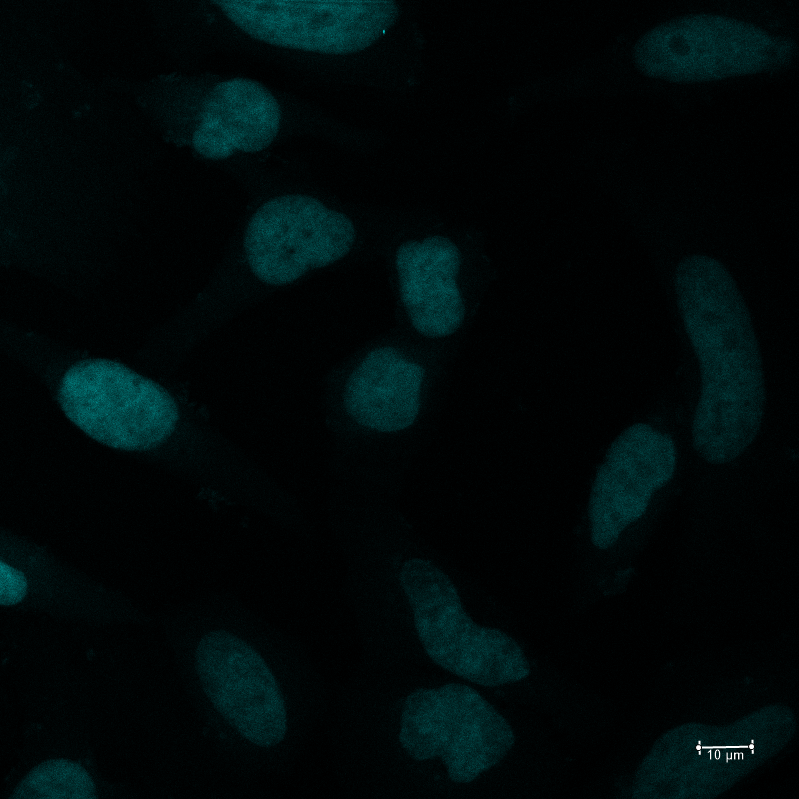

Supplement: Supplementary file 4 — Source data Fig. 3 [file 44318_2024_125_MOESM4_ESM.zip › Figure 3 - Source Data/3C/YFP-ev x PARP9 x ADPr - DAPI.tif]

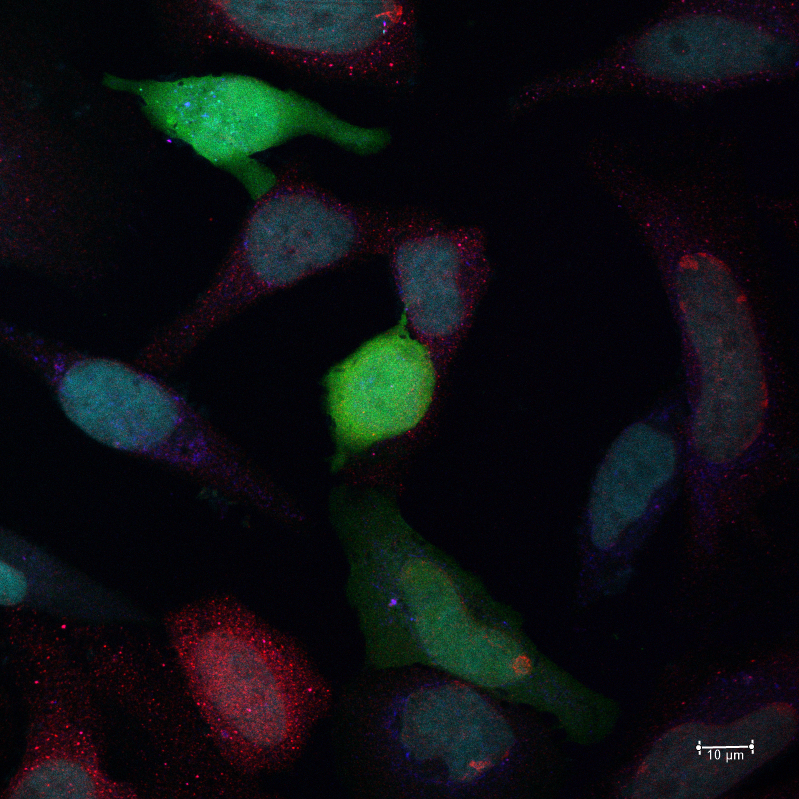

Supplement: Supplementary file 4 — Source data Fig. 3 [file 44318_2024_125_MOESM4_ESM.zip › Figure 3 - Source Data/3C/YFP-ev x PARP9 x ADPr - merge.tif]

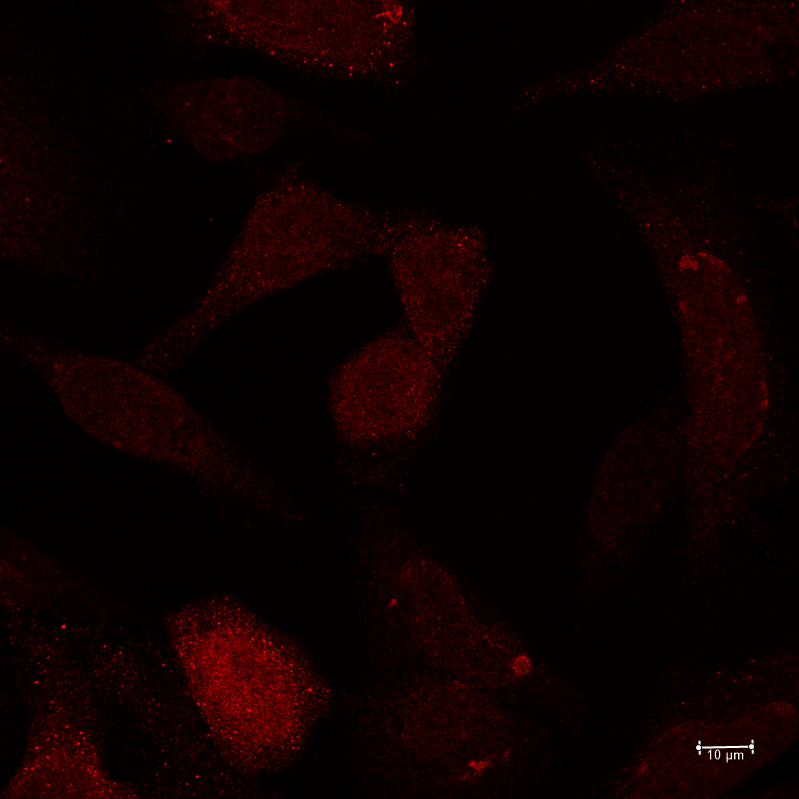

Supplement: Supplementary file 4 — Source data Fig. 3 [file 44318_2024_125_MOESM4_ESM.zip › Figure 3 - Source Data/3C/YFP-ev x PARP9 x ADPr - PARP9.tif]

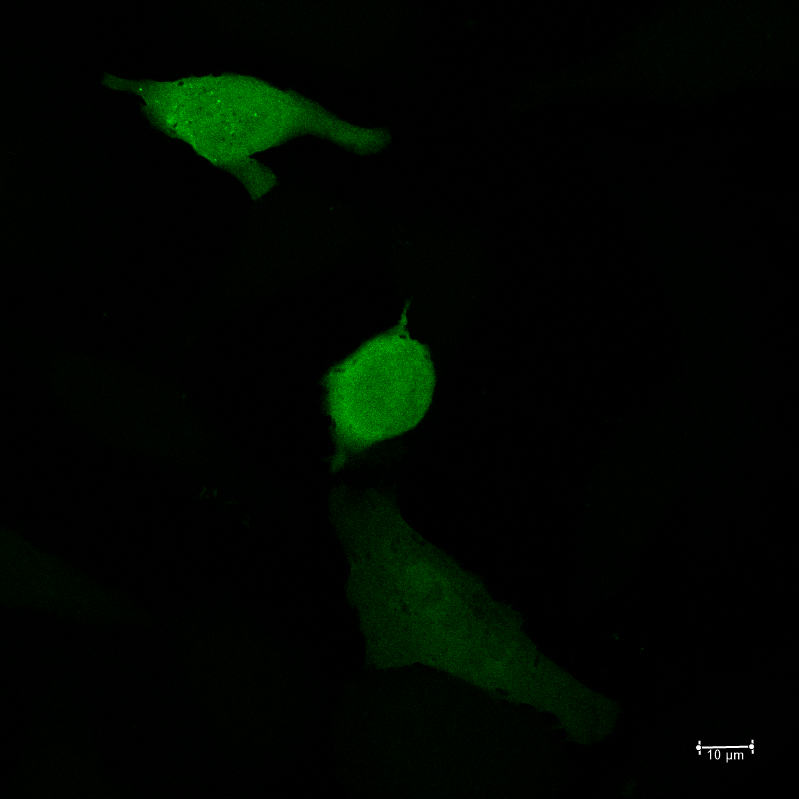

Supplement: Supplementary file 4 — Source data Fig. 3 [file 44318_2024_125_MOESM4_ESM.zip › Figure 3 - Source Data/3C/YFP-ev x PARP9 x ADPr - YFP.tif]

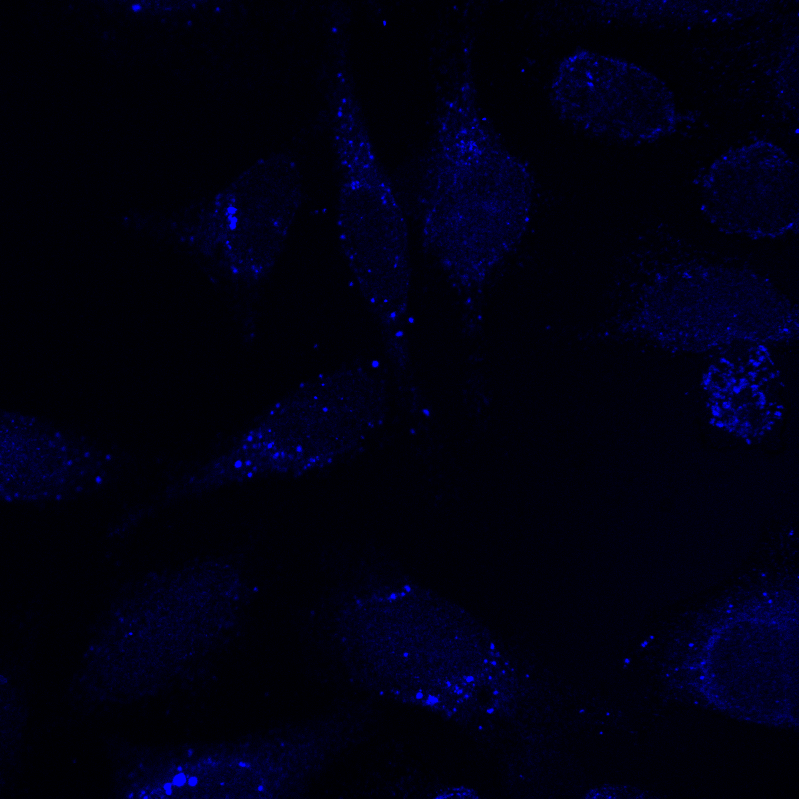

Supplement: Supplementary file 4 — Source data Fig. 3 [file 44318_2024_125_MOESM4_ESM.zip › Figure 3 - Source Data/3C/YFP-PARP14 x PARP9 x ADPr - ADPr.tif]

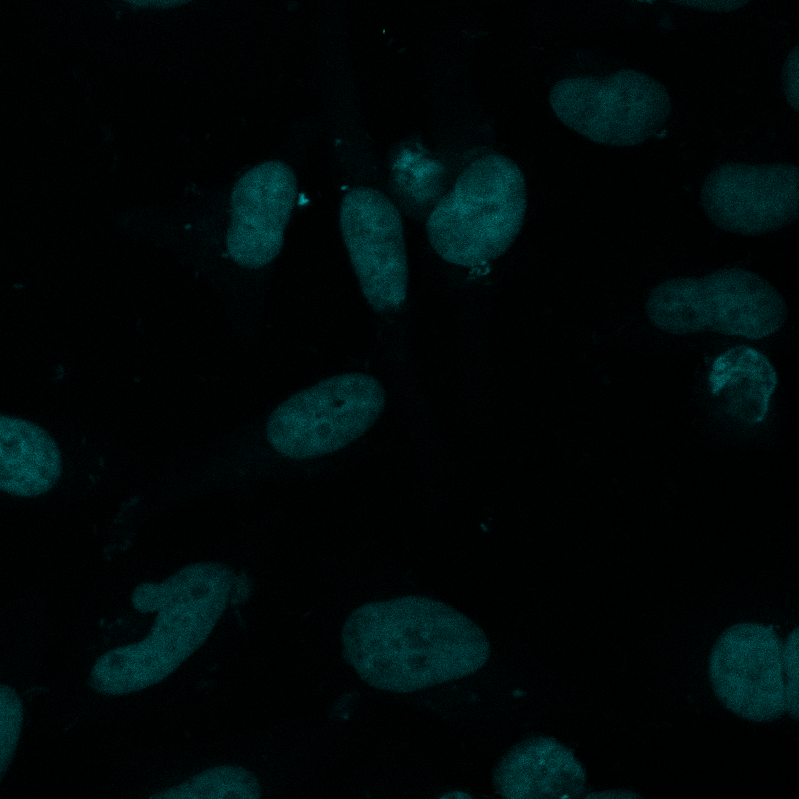

Supplement: Supplementary file 4 — Source data Fig. 3 [file 44318_2024_125_MOESM4_ESM.zip › Figure 3 - Source Data/3C/YFP-PARP14 x PARP9 x ADPr - DAPI.tif]

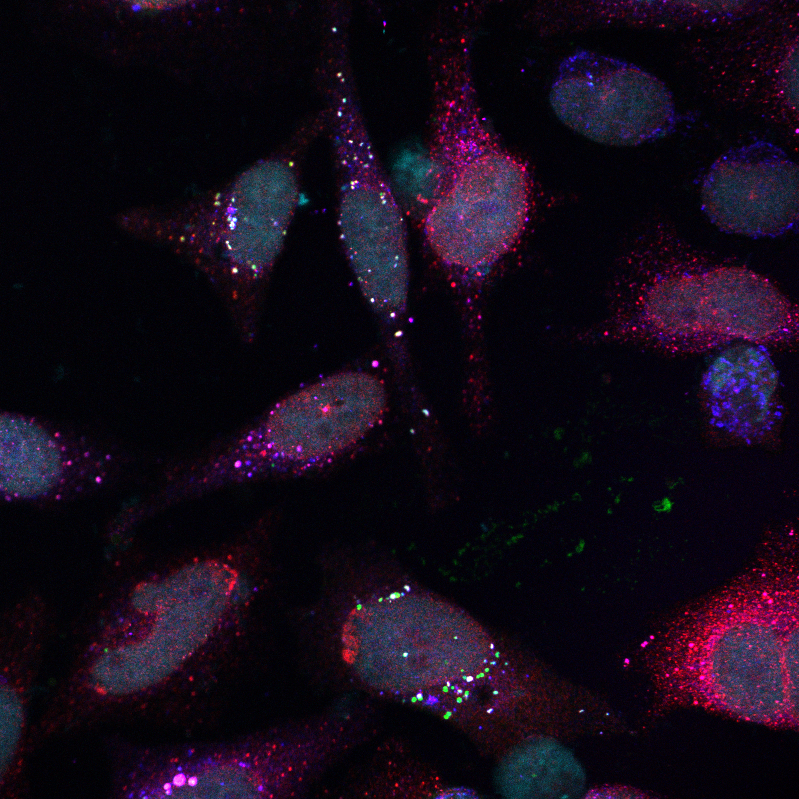

Supplement: Supplementary file 4 — Source data Fig. 3 [file 44318_2024_125_MOESM4_ESM.zip › Figure 3 - Source Data/3C/YFP-PARP14 x PARP9 x ADPr - merge.tif]

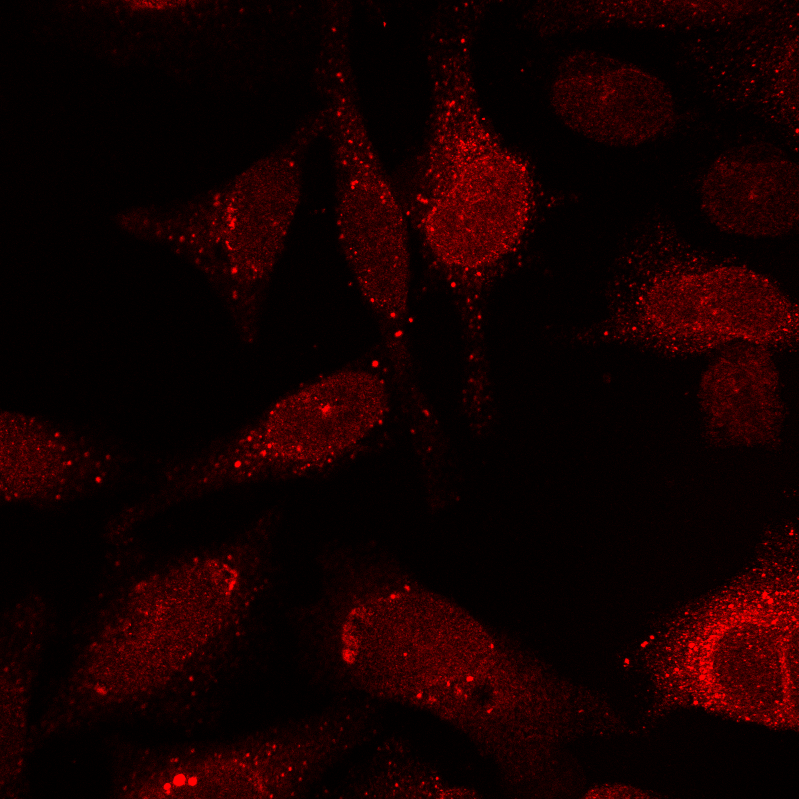

Supplement: Supplementary file 4 — Source data Fig. 3 [file 44318_2024_125_MOESM4_ESM.zip › Figure 3 - Source Data/3C/YFP-PARP14 x PARP9 x ADPr - PARP9.tif]

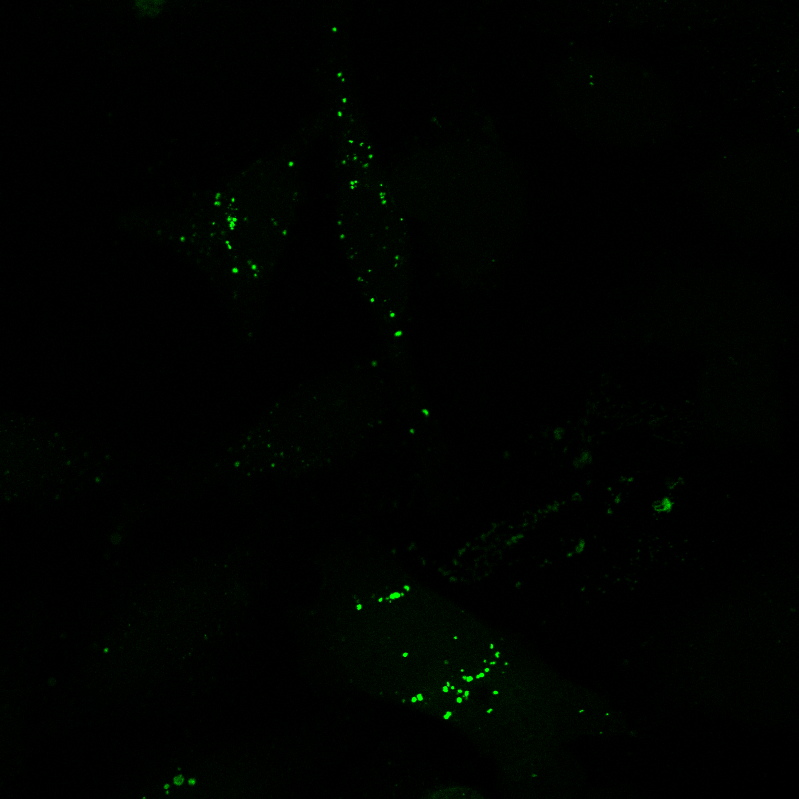

Supplement: Supplementary file 4 — Source data Fig. 3 [file 44318_2024_125_MOESM4_ESM.zip › Figure 3 - Source Data/3C/YFP-PARP14 x PARP9 x ADPr - YFP-PARP14.tif]

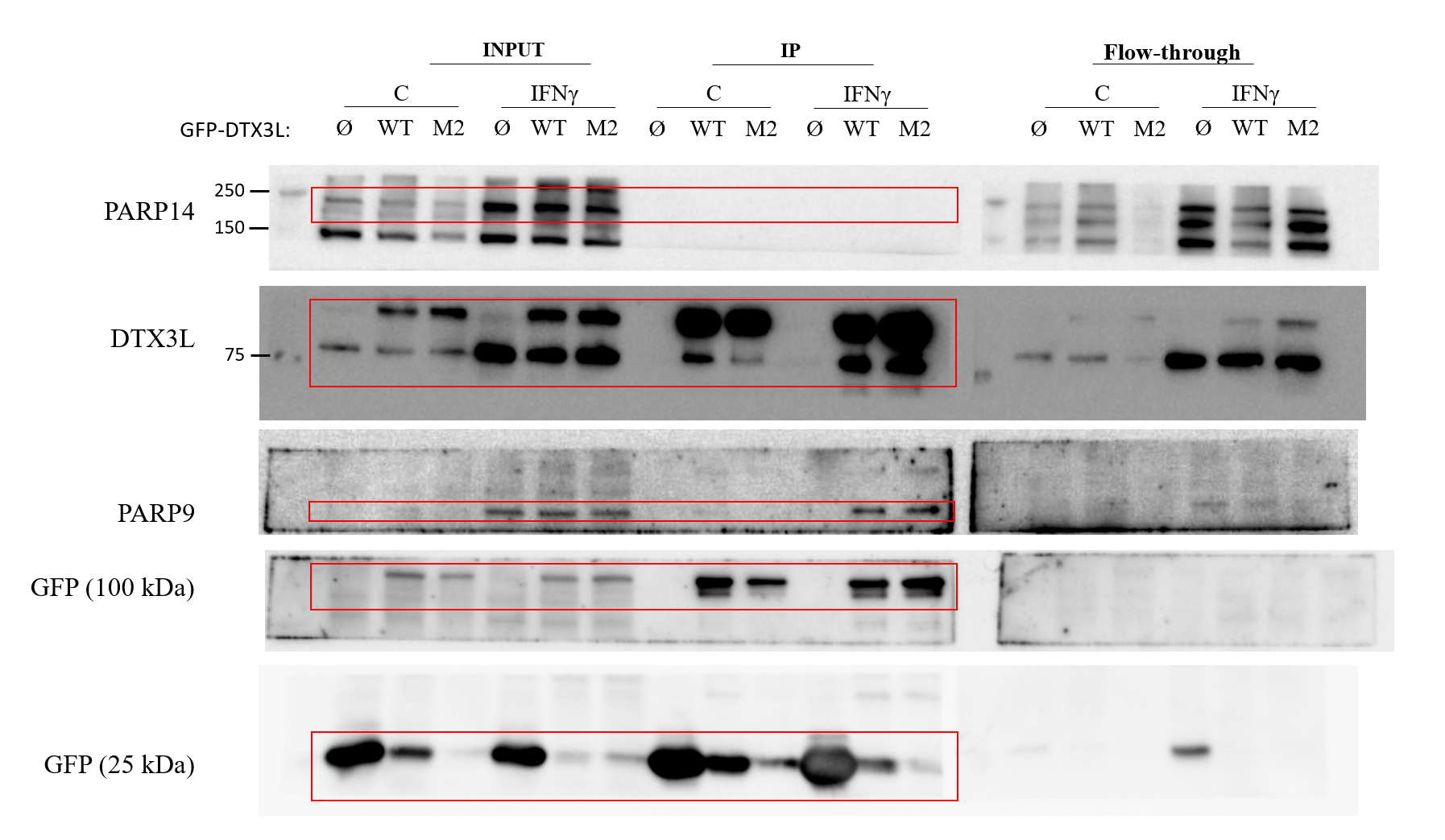

Supplement: Supplementary file 4 — Source data Fig. 3 [file 44318_2024_125_MOESM4_ESM.zip › Figure 3 - Source Data/3D/3D - SD - western blot.tif]

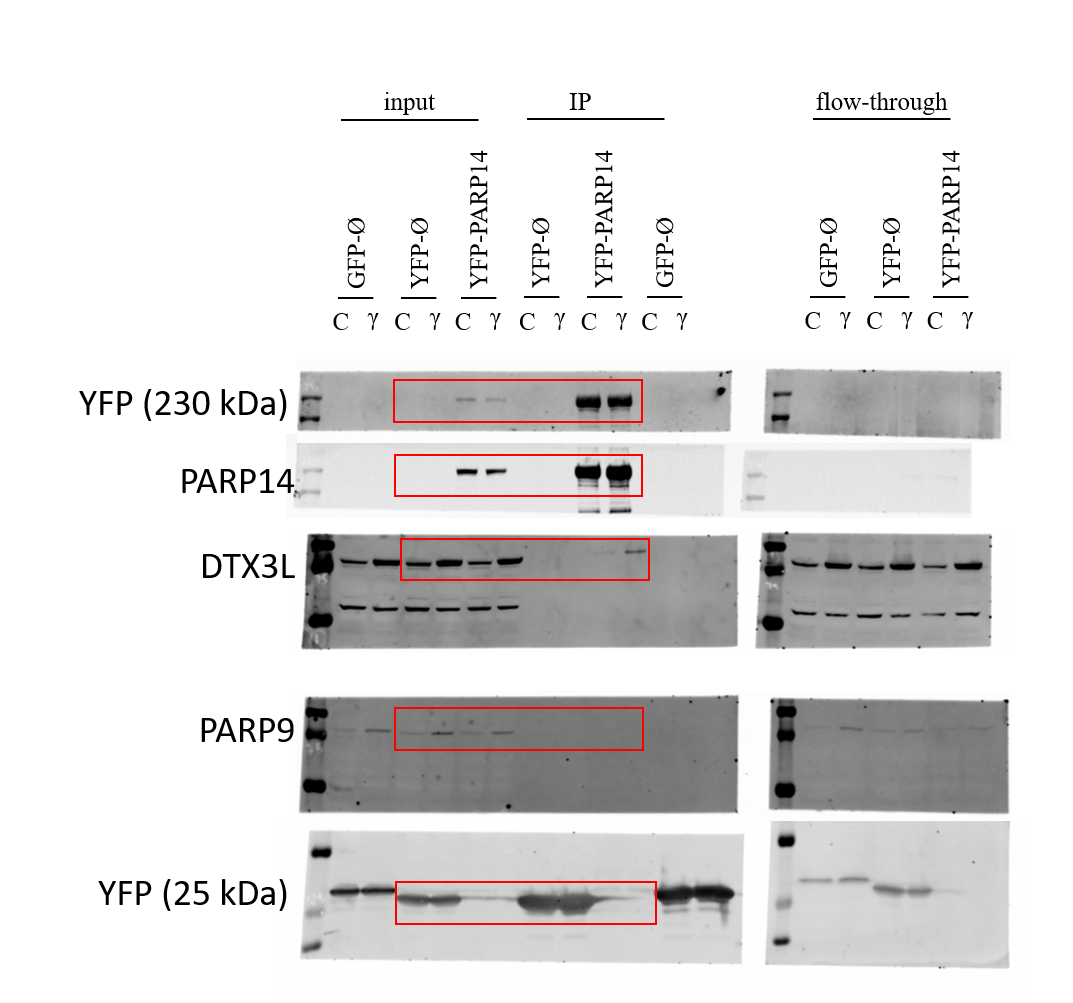

Supplement: Supplementary file 4 — Source data Fig. 3 [file 44318_2024_125_MOESM4_ESM.zip › Figure 3 - Source Data/3E/3E - SD - western blot.tif]

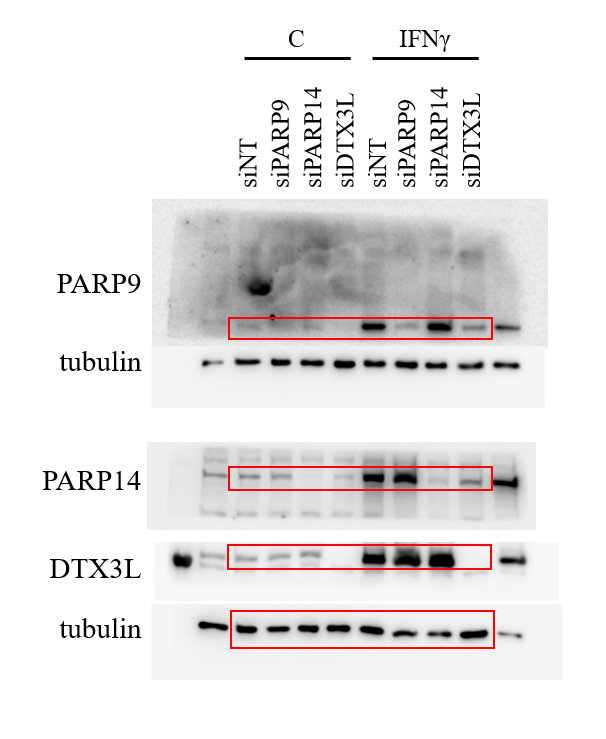

Supplement: Supplementary file 5 — Source data Fig. 4 [file 44318_2024_125_MOESM5_ESM.zip › Figure 4 - Source Data/4A/4A - SD - western blots siPARPs.tif]

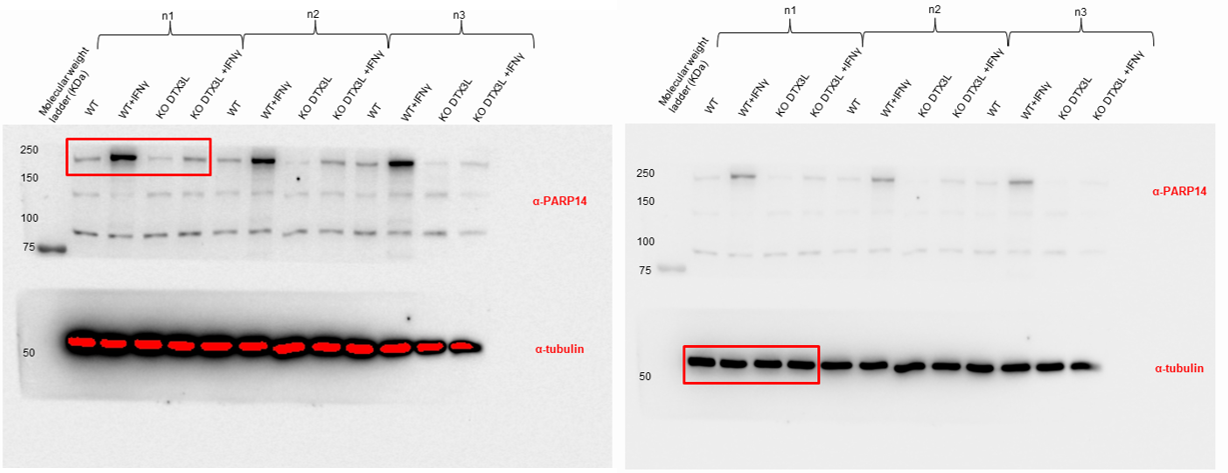

Supplement: Supplementary file 5 — Source data Fig. 4 [file 44318_2024_125_MOESM5_ESM.zip › Figure 4 - Source Data/4B/4B - SD - western blot PARP14 and tubulin.tif]

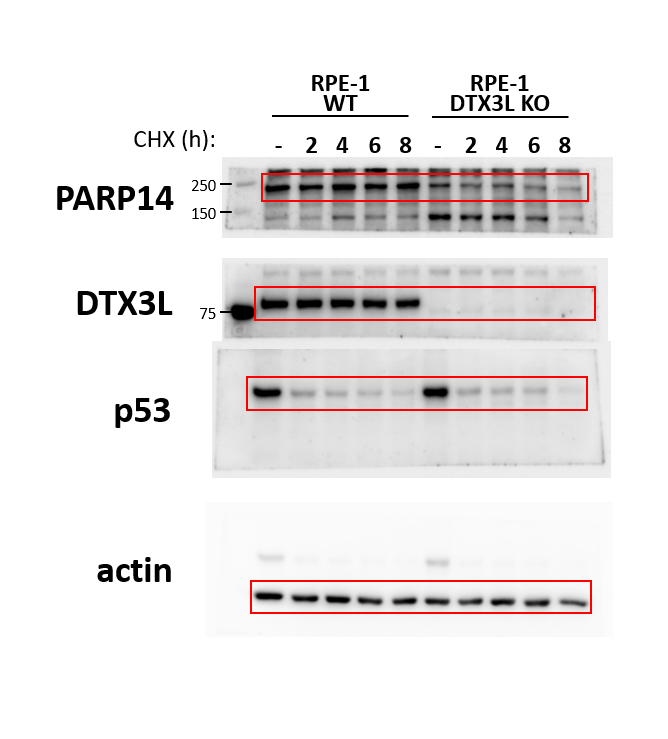

Supplement: Supplementary file 5 — Source data Fig. 4 [file 44318_2024_125_MOESM5_ESM.zip › Figure 4 - Source Data/4D/4D - SD - western blots.tif]

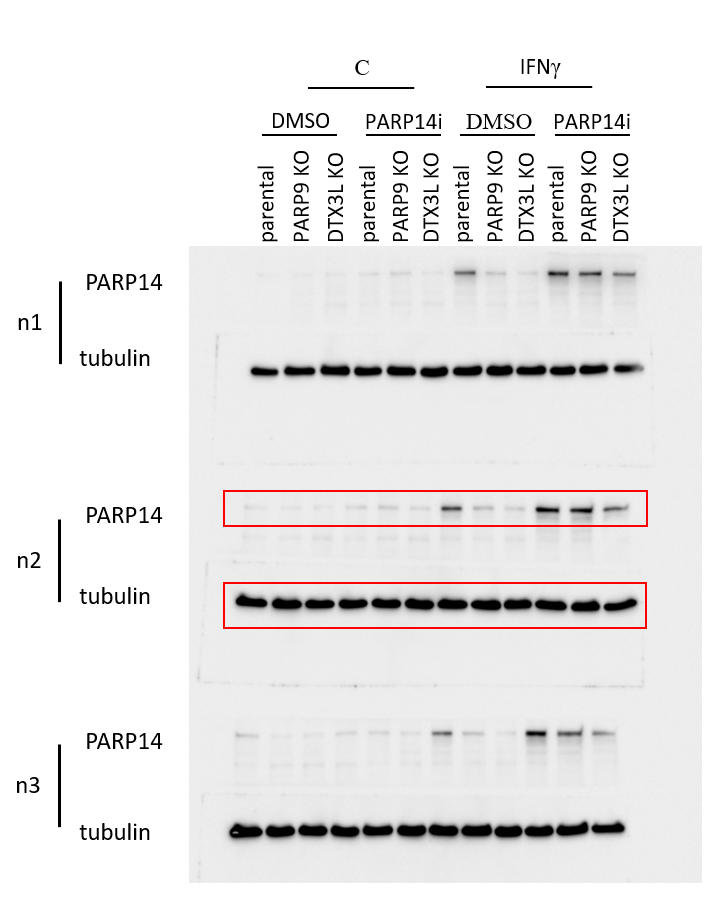

Supplement: Supplementary file 5 — Source data Fig. 4 [file 44318_2024_125_MOESM5_ESM.zip › Figure 4 - Source Data/4E/SD - 4E - western blots.tif]

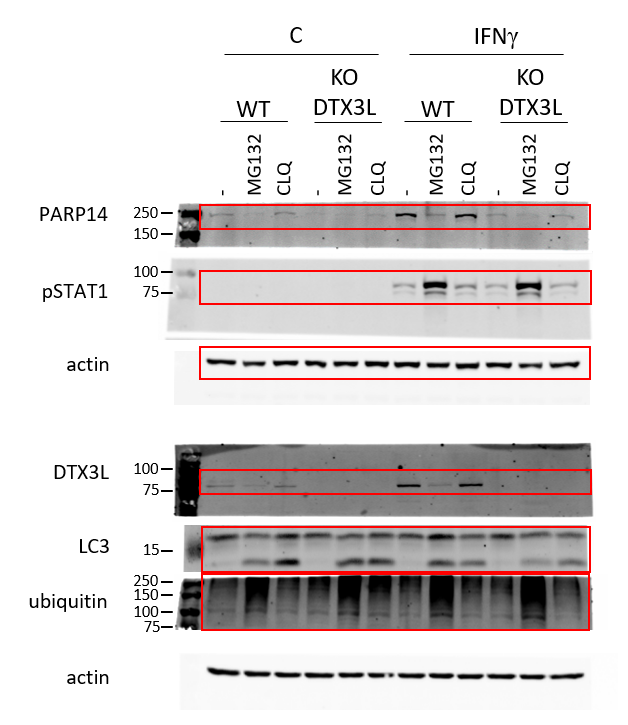

Supplement: Supplementary file 5 — Source data Fig. 4 [file 44318_2024_125_MOESM5_ESM.zip › Figure 4 - Source Data/4F/4F - SD - western blots.tif]

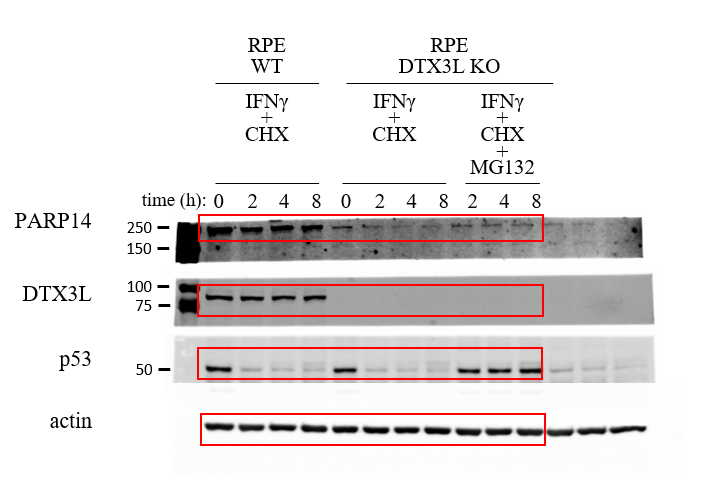

Supplement: Supplementary file 5 — Source data Fig. 4 [file 44318_2024_125_MOESM5_ESM.zip › Figure 4 - Source Data/4G/4G - SD - western blot.tif]

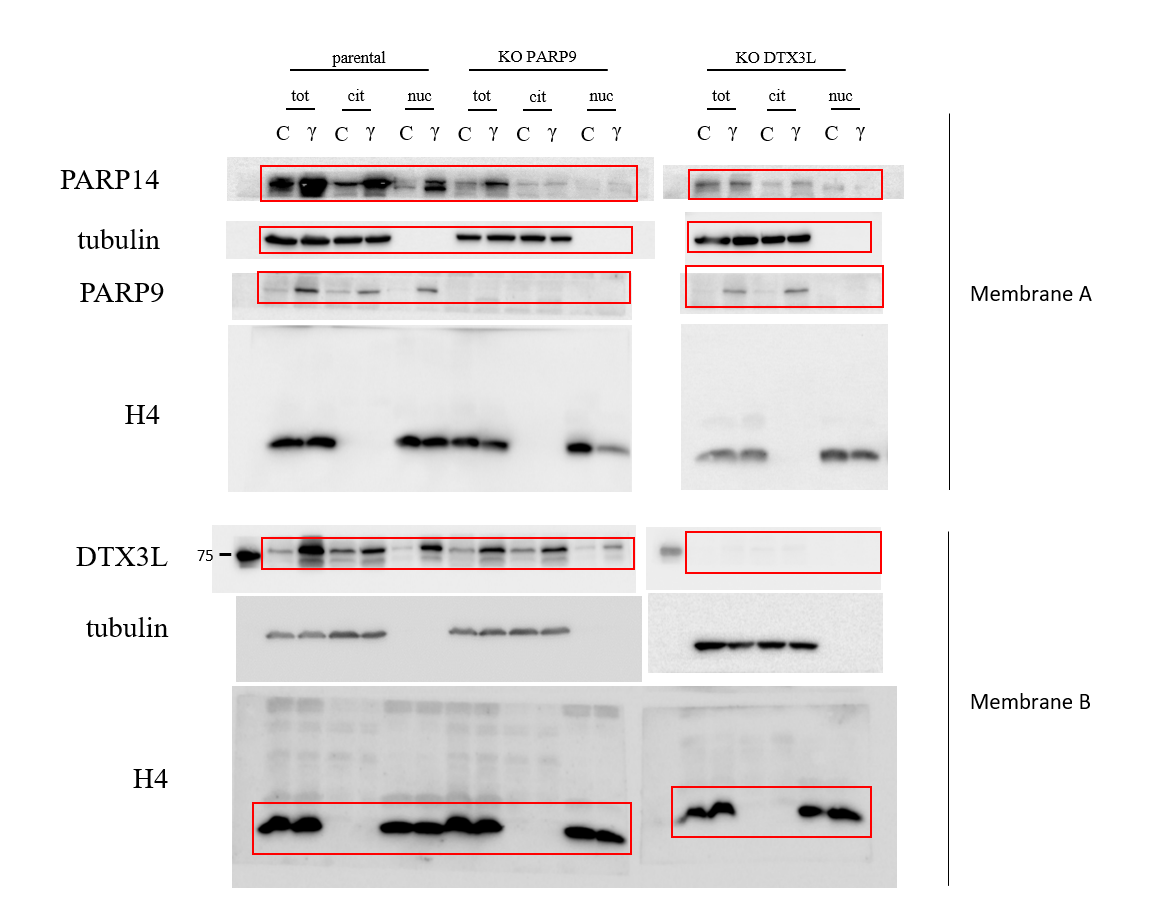

Supplement: Supplementary file 5 — Source data Fig. 4 [file 44318_2024_125_MOESM5_ESM.zip › Figure 4 - Source Data/4H/4H - SD - western blot.tif]

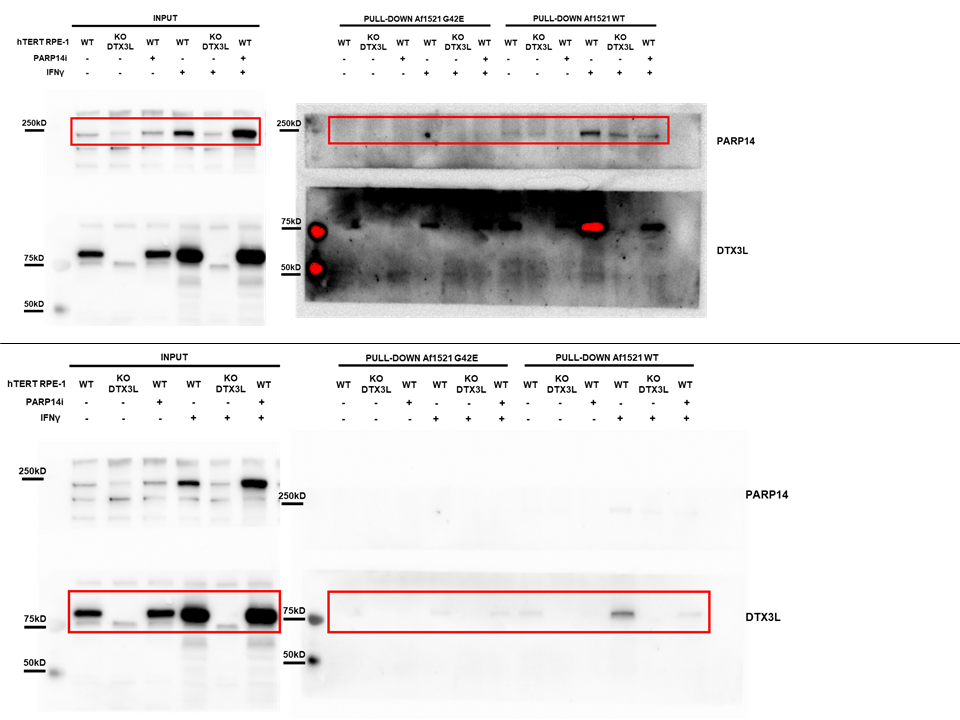

Supplement: Supplementary file 6 — Source data Fig. 5 [file 44318_2024_125_MOESM6_ESM.zip › Figure 5 - Source Data/5A/SOURCE DATA FIG. 5A - western PARP14 and DTX3L.tif]

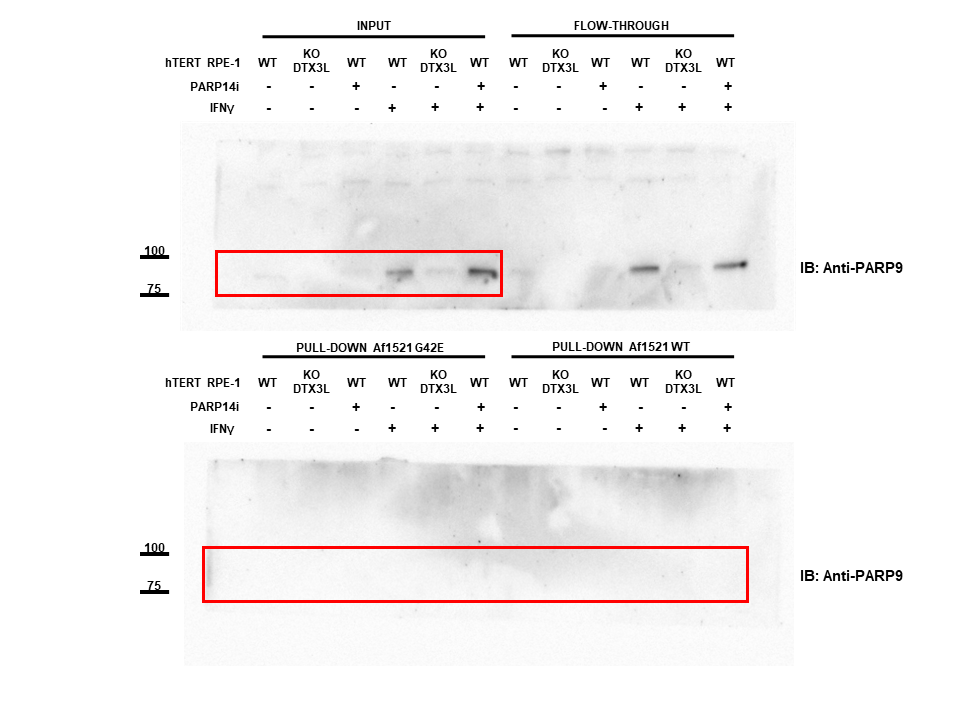

Supplement: Supplementary file 6 — Source data Fig. 5 [file 44318_2024_125_MOESM6_ESM.zip › Figure 5 - Source Data/5A/SOURCE DATA FIG. 5A - western PARP9.tif]

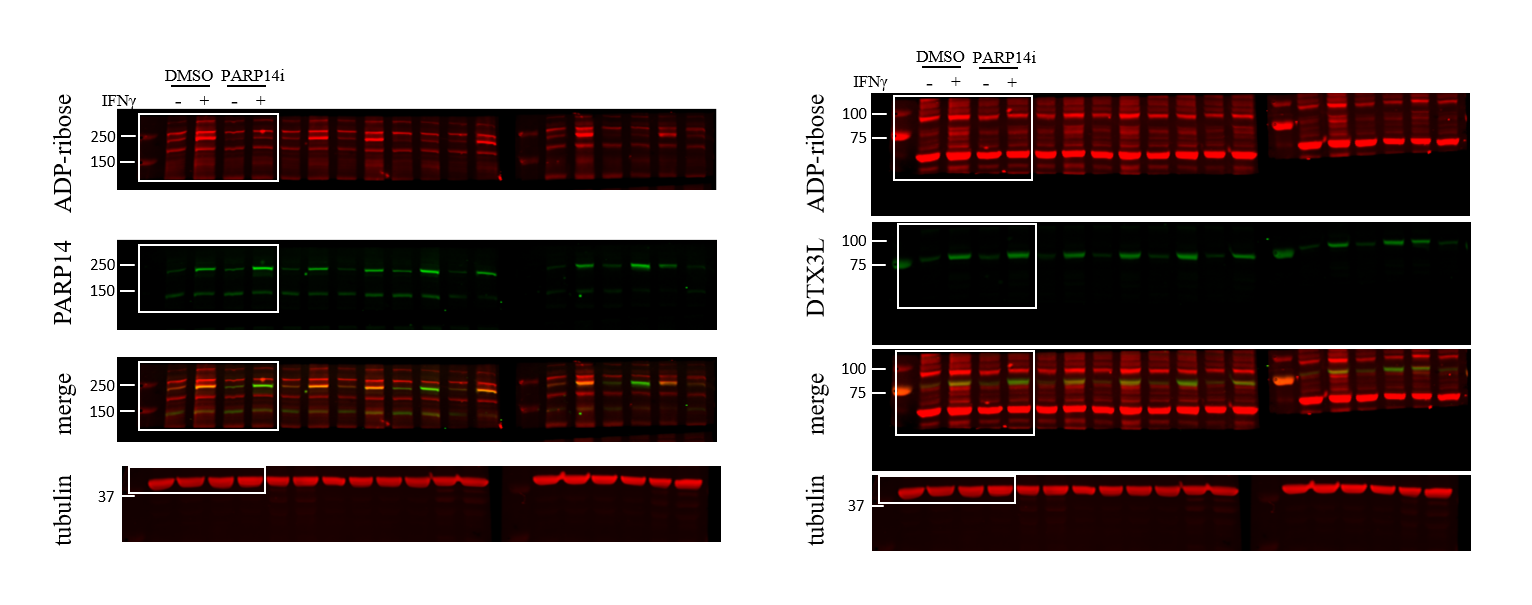

Supplement: Supplementary file 6 — Source data Fig. 5 [file 44318_2024_125_MOESM6_ESM.zip › Figure 5 - Source Data/5B/SD - 5B - blotting fluorescente.tif]

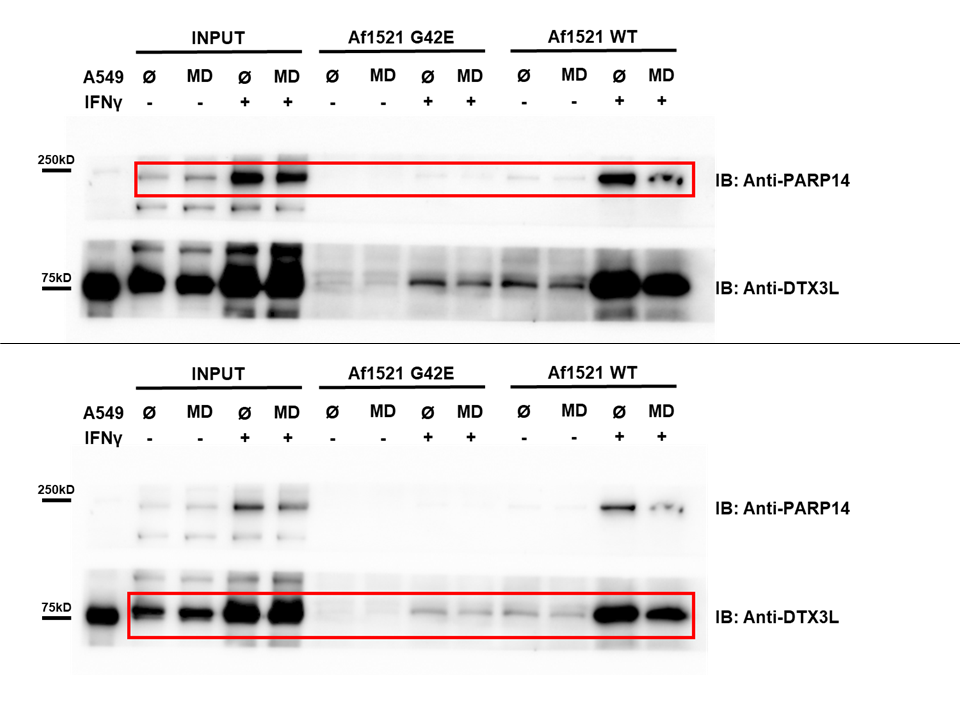

Supplement: Supplementary file 6 — Source data Fig. 5 [file 44318_2024_125_MOESM6_ESM.zip › Figure 5 - Source Data/5C/SOURCE DATA FIG. 5C - western PARP14 and DTX3L.tif]
