# Supplementary material for: PARP14 is regulated by the PARP9/DTX3L complex and promotes interferon γ-induced ADP-ribosylation
Source: EMBO J. 2024 Jun 4;43(14):6. doi: 10.1038/s44318-024-00125-1 (PMC11251048; doi:10.1038/s44318-024-00125-1)
Supplement: Supplementary file 7 — Expanded View Figures [file 44318_2024_125_MOESM7_ESM.pdf]

Expanded View Figures

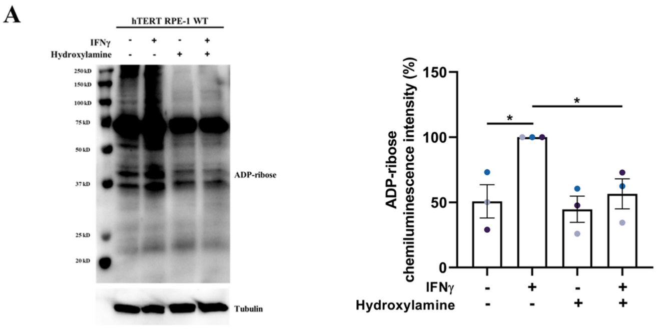

Figure EV1. IFNγ-induced ADP-ribosylation is hydroxylamine-sensitive.

(related to figure 1). (**A**) Representative image (left) and quantification (right) of immunoblot analyses for mono-ADP-ribose (43647 HRP-coupled) levels relative to tubulin loading control in RPE-1 cells treated with vehicle control or 200 U/mL IFNγ for 24 h. After cell lysis, indicated samples were incubated with 1 M hydroxylamine pH 7.0 for 1 h. For quantification, the 75 kDa saturated band was excluded from analysis and the signal intensity relative to tubulin loading control was normalised to IFNγ-treated cells. Mean ± SEM ($n = 3$, from three separate experiments). *$p < 0.05$.

 © The Author(s)

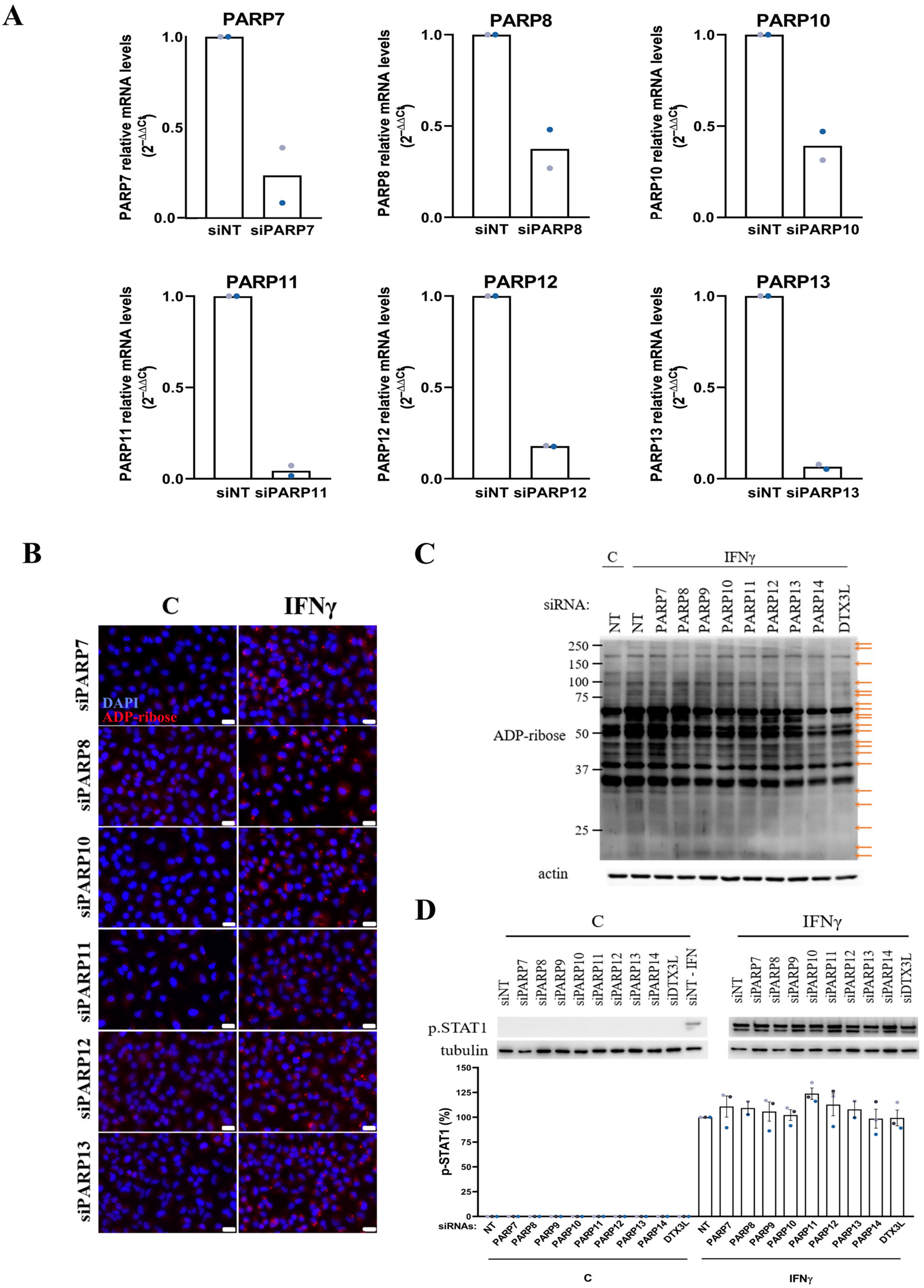
A
PARP7
PARP7 relative mRNA levels (2^-ΔΔCt)
siNT siPARP7
PARP8
PARP8 relative mRNA levels (2^-ΔΔCt)
siNT siPARP8
PARP10
PARP10 relative mRNA levels (2^-ΔΔCt)
siNT siPARP10
PARP11
PARP11 relative mRNA levels (2^-ΔΔCt)
siNT siPARP11
PARP12
PARP12 relative mRNA levels (2^-ΔΔCt)
siNT siPARP12
PARP13
PARP13 relative mRNA levels (2^-ΔΔCt)
siNT siPARP13
1.0
0.5
0.0
B
C
IFNγ
siPARP7
siPARP8
siPARP10
siPARP11
siPARP12
siPARP13
DAPI
ADP-ribose
C
C
IFNγ
siRNA:
NT NT PARP7 PARP8 PARP9 PARP10 PARP11 PARP12 PARP13 PARP14 DTX3L
250
150
100
75
50
37
25
ADP-ribose
actin
D
C
IFNγ
siNT siPARP7 siPARP8 siPARP9 siPARP10 siPARP11 siPARP12 siPARP13 siPARP14 siDTX3L siNT - IFN
siNT siPARP7 siPARP8 siPARP9 siPARP10 siPARP11 siPARP12 siPARP13 siPARP14 siDTX3L
p.STAT1
tubulin
p-STAT1 (%)
150
125
100
75
50
25
0
siRNAs:
NT PARP7 PARP8 PARP9 PARP10 PARP11 PARP12 PARP13 PARP14 DTX3L
NT PARP7 PARP8 PARP9 PARP10 PARP11 PARP12 PARP13 PARP14 DTX3L
C
IFNγ

Figure EV2. IFNγ-induced ADP-ribosylation is dependent on the PARP9/DTX3L complex and PARP14.

(related to figure 2). (**A**) Quantification of relative PARP7, PARP8, PARP10, PARP11, PARP12 and PARP13 mRNA levels by RT-qPCR in A549 cells transfected with the indicated siRNAs, normalised to siNT transfected cells. Mean ± SEM (*n* = 2, from two separate experiments). siRNA efficiencies for PARP9, DTX3L and PARP14 are shown in Fig. 4A (**B**) Representative immunofluorescence microscopy images of pan-ADP-ribose (MABE1016) signal in A549 cells transfected with the indicated siRNAs, treated with vehicle control or 100 U/mL IFNγ for 24 h. Scale bar: 20 µm. siNT control is shown in Fig. 2A (**C**) Representative immunoblot for mono-ADP-ribose (43647 HRP-coupled) and actin loading control in A549 cells transfected with the indicated siRNAs, treated with vehicle control or 200 U/mL IFNγ for 24 h. (**D**) Representative images (upper) and quantification (lower) of immunoblot analyses for STAT1 phospho-Y701 (p.STAT1) levels relative to tubulin loading control in A549 cells transfected with the indicated siRNAs, treated with vehicle control or 100 U/mL IFNγ for 24 h, normalised to IFNγ-treated siNT cells. Mean ± SEM (*n* = 3, from three separate experiments).

© The Author(s)

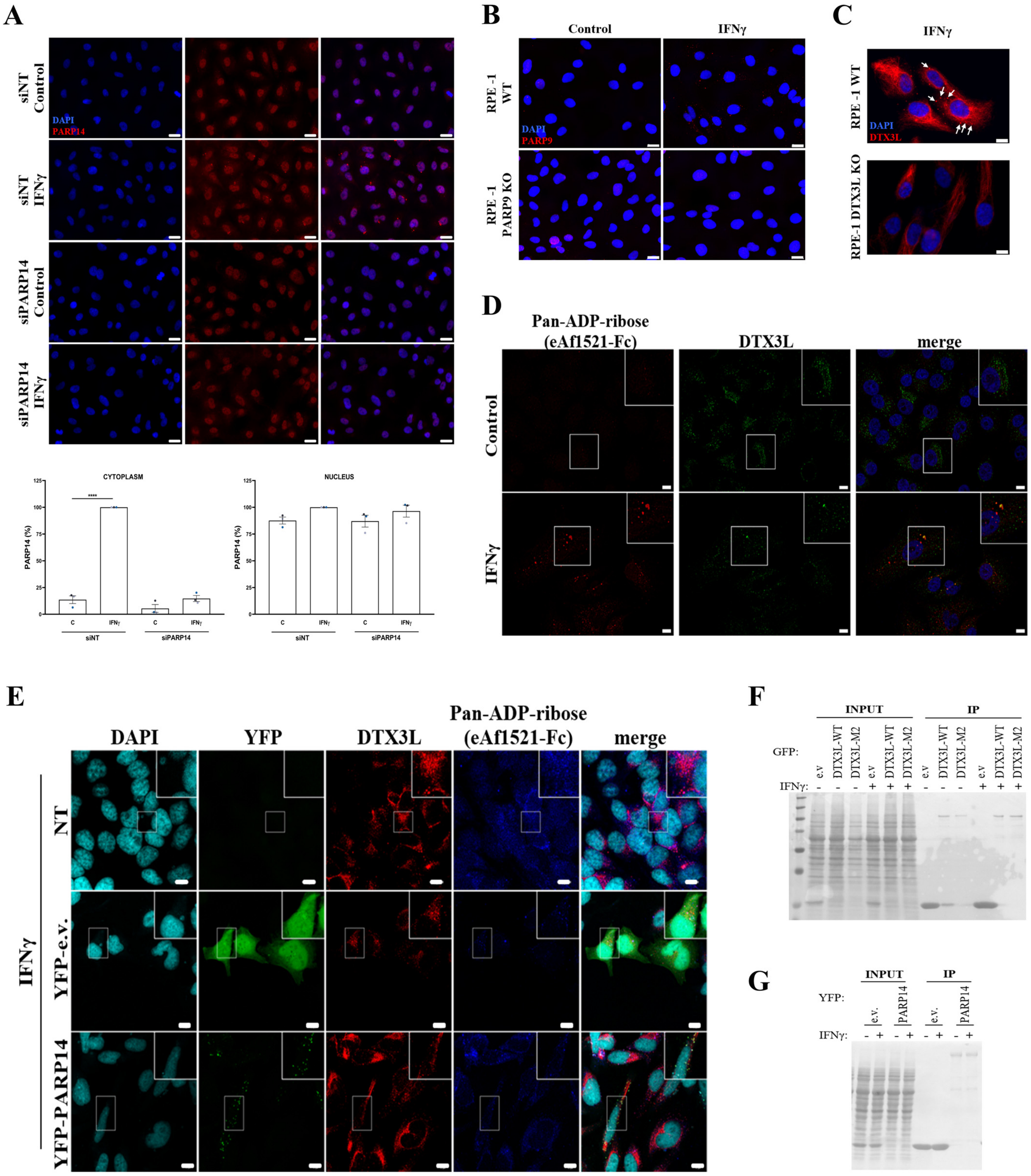

A
siNT Control
siNT IFNγ
siPARP14 Control
siPARP14 IFNγ
DAPI
PARP14
CYTOPLASM
NUCLEUS
PARP14 (%)
C
IFNγ
siNT
siPARP14
B
Control
IFNγ
RPE -1 WT
RPE -1 PARP9 KO
DAPI
PARP9
C
IFNγ
RPE -1 WT
RPE-1 DTX3L KO
DAPI
DTX3L
D
Pan-ADP-ribose (eAf1521-Fc)
DTX3L
merge
Control
IFNγ
E
DAPI
YFP
DTX3L
Pan-ADP-ribose (eAf1521-Fc)
merge
IFNγ
NT
YFP-e.v.
YFP-PARP14
F
INPUT
IP
GFP:
e.v
DTX3L-WT
DTX3L-M2
IFNγ:
G
INPUT
IP
YFP:
e.v.
PARP14
IFNγ:

© The Author(s)

Figure EV3. Antibody validation, IP controls and co-localization of DTX3L with PARP14 and ADP-ribose.

(related to figure 3). (**A–C**) PARP14, PARP9 and DTX3L antibody validation. (**A**) Representative immunofluorescence microscopy images (upper) and quantification (lower) of PARP14 signal in the cytoplasm (lower left) and nuclei (lower right) in A549 cells transfected with indicated siRNAs, treated with vehicle control or 100 U/mL IFNγ for 24 h. (**B**) Representative immunofluorescence microscopy images of PARP9 staining in RPE-1 WT or PARP9 knockout RPE-1 cells treated with vehicle control or 100 U/mL IFNγ for 24 h. (**C**) Representative immunofluorescence microscopy images of DTX3L staining in RPE-1 WT or DTX3L knockout RPE-1 cells treated with 100 U/mL IFNγ for 24 h. White arrows indicate the specific DTX3L cytoplasmic dots. Scale bar: 10 µm. (**D**) Representative immunofluorescence microscopy images of A549 cells treated or not with 500 U/mL IFNγ for 24 h, co-stained for pan-ADP-ribose (eAF1521-Fc) and DTX3L. Regions marked with a white box are enlarged in the top right corner. Scale bar: 10 µm. (**E**) Representative immunofluorescence confocal microscopy images of HeLa cells not transfect (NT) or transfected with YFP-empty vector (YFP-e.v.) or YFP-PARP14, treated with 200 U/mL IFNγ for 24 h, co-stained for pan-ADP-ribose (eAF1521-Fc) and DTX3L. Regions marked with a white box are enlarged in the top right corner. Scale bar: 10 µm. (**F**, **G**) Ponceau S staining of membranes to confirm equal loading and transfer of proteins used in Fig. 3D (**F**) and 3E (**G**).

© The Author(s)

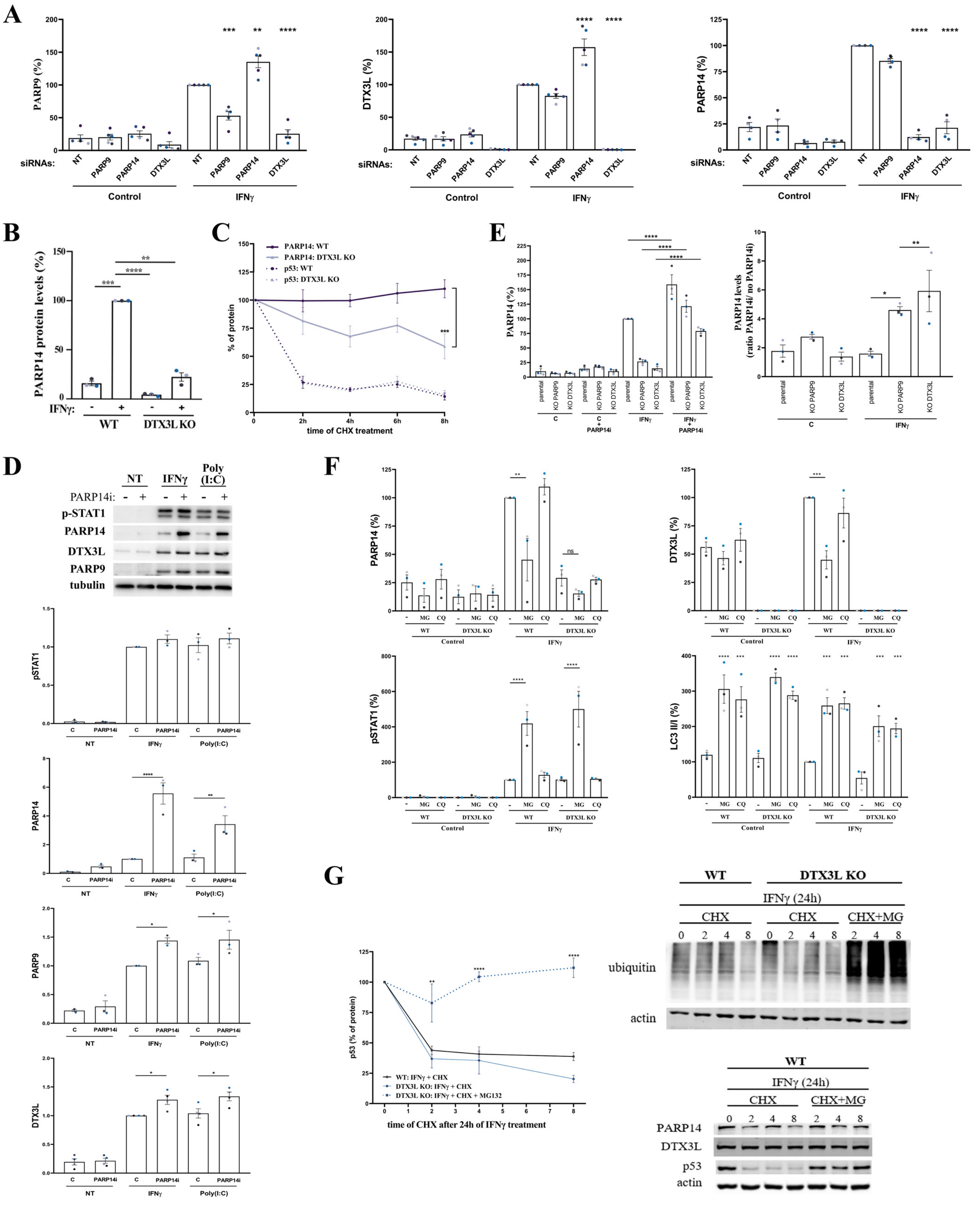
A
PARP9 (%)
DTX3L (%)
PARP14 (%)
siRNAs: NT PARP9 PARP14 DTX3L NT PARP9 PARP14 DTX3L
Control
IFNγ
B
PARP14 protein levels (%)
IFNγ: - + - +
WT
DTX3L KO
C
PARP14: WT
PARP14: DTX3L KO
p53: WT
p53: DTX3L KO
% of protein
time of CHX treatment
E
PARP14 (%)
parental KO PARP9 KO DTX3L
C
C + PARP14i
IFNγ
IFNγ + PARP14i
PARP14 levels (ratio PARP14i/ no PARP14i)
D
NT IFNγ Poly (I:C)
PARP14i: - + - + - +
p-STAT1
PARP14
DTX3L
PARP9
tubulin
pSTAT1
PARP14
PARP9
DTX3L
C PARP14i
NT IFNγ Poly(I:C)
F
PARP14 (%)
DTX3L (%)
pSTAT1 (%)
LC3 II/I (%)
- MG CQ
WT DTX3L KO
Control IFNγ
G
WT DTX3L KO
IFNγ (24h)
CHX CHX CHX+MG
0 2 4 8 0 2 4 8 2 4 8
ubiquitin
actin
p53 (% of protein)
WT: IFNγ + CHX
DTX3L KO: IFNγ + CHX
DTX3L KO: IFNγ + CHX + MG132
time of CHX after 24h of IFNγ treatment
WT
IFNγ (24h)
CHX CHX+MG
0 2 4 8 2 4 8
PARP14
DTX3L
p53
actin

Figure EV4. The PARP9/DTX3L complex regulates PARP14 protein stability.

(related to figure 4). (**A**) Quantification of immunoblot analyses (as shown in Fig. 4A) for PARP9, DTX3L and PARP14 protein levels relative to tubulin loading control in A549 cells transfected with indicated siRNAs and treated with vehicle control or 100 U/ml IFNγ for 24 h, normalised to IFNγ-treated siNT cells. (**B**) Quantification of immunoblot analyses (as shown in Fig. 4B) for PARP14 protein levels relative to tubulin loading control in RPE-1 WT or DTX3L KO cells treated with vehicle control or 100 U/mL IFNγ for 24 h, normalised to IFNγ-treated WT cells. (**C**) Quantification of immunoblot analyses (as shown in Fig. 4D) for PARP14 and p53 in RPE-1 WT or DTX3L KO cells treated with 50 μg/mL cycloheximide (CHX) for the indicated times, normalised to untreated controls. (**D**) Representative image and quantification of immunoblot analyses for pSTAT1, PARP14, PARP9 and DTX3L levels relative to tubulin loading control in A549 cells, 24 h after treatment with vehicle control, 100 U/mL IFNγ, transfection with 0.1 μg/mL poly(I:C) and/or 100 nM PARP14i, normalised to IFNγ-treated cells. (**E**) Quantification of immunoblot analyses (as shown in Fig. 4E) for PARP14 protein in RPE-1 WT, PARP9 KO or DTX3L KO cells treated with vehicle control or 100 U/mL IFNy and/or 100 nM PARP14i for 24 h, as indicated. PARP14 levels relative to tubulin loading control, normalised to IFNγ-treated WT cells (left) and the ratio between the PARP14i treated and respective non-treated samples (right) are shown. (**F**) Quantification of immunoblot analyses (shown in Fig. 4F) for PARP14, DTX3L, STAT1 phospho-Y701 (p-STAT1) levels and LC3II/LC3I ratio relative to tubulin loading control in RPE-1 WT or DTX3L KO cells treated with vehicle controls or 100 U/mL IFNy, and 20 μM chloroquine (CQ) or 10 μM MG132 as indicated, for 24 h, normalised to IFNγ-treated WT cells. (**G**) Quantification of immunoblot analyses for p53 in RPE-1 WT or DTX3L KO (left) and representative image of immunoblot analyses for ubiquitin (upper right) and PARP14, DTX3L, p53 and actin loading control (lower right) in RPE-1 WT cells treated with 50 μg/mL cycloheximide (CHX) and 10 μM MG132 for the indicated times after treatment with 100 U/mL IFNγ for 24 h, normalised to untreated samples of each cell line. Mean ± SEM ($n = 3$–5, as indicated). *$p < 0.05$, **$p < 0.01$, ***$p < 0.001$ and ****$p < 0.0001$.

 © The Author(s)

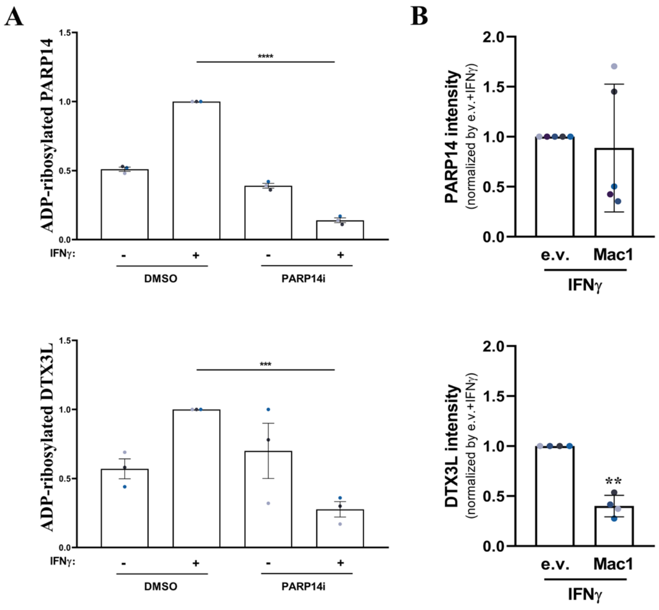

Figure EV5. ADP-ribosylation of PARP14 and DTX3L in response to IFNγ is sensitive to Mac1 expression.

(related to figure 5). (**A**) Quantification of immunoblot analyses for fluorescently co-stained mono-ADP-ribose (43647 mouse Fc-conjugated) and either PARP14 (upper) or DTX3L (lower) in A549 cells treated with vehicle control or 100 U/mL IFNγ for 24 h and/or 100 nM PARP14 inhibitor, as indicated. Graphs show the ratio between the mono-ADP-ribose band at the respective molecular weight and the total protein levels, normalised to the IFNγ-treated sample. (**B**) Quantification of PARP14 (upper) and DTX3L (left) immunoblot bands of GST-*Af1521* pulldown in A549 cells transduced with an empty vector (e.v.) or FLAG-tagged SARS-CoV-2 Nsp3 macrodomain (Mac1) lysates, 24 h after treatment with 100 U/mL IFNγ. Mean ± SEM ($n = 3$–5, as indicated). ***$p < 0.001$ and ****$p < 0.0001$.

© The Author(s)
